# Healthcare Workers’ Attitudes towards Mandatory COVID-19 Vaccination: A Systematic Review and Meta-Analysis

**DOI:** 10.3390/vaccines11040880

**Published:** 2023-04-21

**Authors:** Marios Politis, Sotiris Sotiriou, Chrysoula Doxani, Ioannis Stefanidis, Elias Zintzaras, Georgios Rachiotis

**Affiliations:** 1 Department of Biomathematics, School of Medicine, University of Thessaly, 41222 Larissa, Greece; 2 Department of Pathology, Faculty of Medicine, Aristotle University, 54124 Thessaloniki, Greece; 3 Department of Nephrology, School of Medicine, University of Thessaly, 41110 Larissa, Greece; 4 Center for Clinical Evidence Synthesis, Tufts University School of Medicine, Boston, MA 02111, USA; 5 The Institute for Clinical Research and Health Policy Studies, Tufts Medical Center, Boston, MA 02111, USA; 6 Department of Hygiene and Epidemiology, School of Medicine, University of Thessaly, 41110 Larissa, Greece

**Keywords:** healthcare workers, mandatory COVID-19 vaccination, publication bias, global health policy

## Abstract

Background: COVID-19 vaccine mandates are considered a controversial public health policy both in public debate and among healthcare workers (HCWs). Thus, the objective of this systematic review is to give a deep insight into HCWs’ views and attitudes towards COVID-19 vaccination mandates amid the ongoing COVID-19 pandemic. Methods: A systematic literature search of five databases (PubMed, Scopus, Embase, CINAHL, and Web of Science) was conducted between July 2022 and November 2022. Original quantitative studies that addressed the attitudes of HCWs regarding COVID-19 vaccine mandates were considered eligible for this systematic review. All the included studies (n = 57) were critically appraised and assessed for risk of systematic bias. Meta-analyses were performed, providing a pooled estimate of HCWs’ acceptance towards COVID-19 vaccine mandates for: 1. HCWs and 2. the general population. Results: In total, 64% (95% CI: 55%, 72%) of HCWs favored COVID-19 vaccine mandates for HCWs, while 50% (95% CI: 38%, 61%) supported mandating COVID-19 vaccines for the general population. Conclusions: Our findings indicate that mandatory vaccination against COVID-19 is a highly controversial issue among HCWs. The present study provides stakeholders and policy makers with useful evidence related to the compulsory or non-compulsory nature of COVID-19 vaccinations for HCWs and the general population. Other: The protocol used in this review is registered on PROSPERO with the ID number: CRD42022350275.

## 1. Introduction

According to the World Health Organization (W.H.O.), policies that encourage voluntary vaccination, such as public information campaigns, should take priority over mandatory vaccination policies [1]. However, COVID-19 vaccine mandates are considered a reasonable measure when other alternatives fail to achieve adequate vaccination coverage, while both strategies can be adopted simultaneously if deemed beneficial. HCWs are often a target population when it comes to vaccine mandates, in part because of their moral obligation to avoid harming their patients, but also because of their important societal role, especially in an emerging pandemic [1]. In November 2021, COVID-19 vaccination mandates for NHS staff were announced, although vaccination rates among NHS trust healthcare staff were 93% and 90% for the first and second doses, respectively [2]. Furthermore, the percentage of unvaccinated NHS staff was close to 5% in January 2022, and no considerable further increase in vaccination coverage was expected with vaccine mandates, jeopardizing the employment of remaining unvaccinated HCWs, and putting additional pressure on an understaffed NHS [3]. In line with this, many other European countries, e.g., Greece and France, introduced COVID-19 vaccination mandates for healthcare personnel, following Italy, which was the first European country to impose COVID-19 vaccine mandates for HCWs [4]. Interestingly, 6–15% of Italian HCWs remained unvaccinated, despite the extreme first wave of COVID-19 that Italy suffered [5]. Furthermore, almost immediately after the launch of the COVID-19 vaccine, only 52% of frontline HCWs were vaccinated in the US, while one-third of unvaccinated HCWs persisted in not receiving the COVID-19 vaccine [6]. In line with many European countries, the USA decided on mandatory vaccination against COVID-19 immediately after the approval of the COVID-19 vaccine by the US Food and Drug Administration [6].

Vaccination hesitancy among HCWs was a public health concern even before the COVID-19 pandemic [7]. Common causes of vaccination hesitancy among HCWs, both before and during the COVID-19 pandemic era, include mistrust of the authorities, waiting for reliable data, and doubts about the safety and efficacy of the vaccine [7]. However, although vaccine hesitancy is a common problem for many vaccines, additional concerns about rapid roll-out and the use of new m-RNA technology are unique features of the COVID-19 vaccine [7]. According to a scoping review with 76,741 participants, the reported global prevalence of COVID-19 vaccine hesitancy among HCWs was 22.51% (from 4.3 to 72%) [8]. Furthermore, HCWs’ vaccine hesitancy contributes to the distortion of their role as protectors of public health [8]. This is particularly important amid an ongoing pandemic, as HCWs are seen as reliable and impactful sources of health-related information [9,10]. In addition, transmission rates among fully vaccinated, not fully vaccinated, and unvaccinated HCWs varied widely, raising concerns about potential transmission from unvaccinated HCWs to their colleagues and healthcare consumers [11]. As a result, prioritizing the protection of public health over individual liberty has, in many cases, been the moral justification for implementing a mandatory vaccination policy for COVID-19 [12].

COVID-19 vaccines have been shown to be effective in reducing both disease spread and the risk of severe clinical outcomes [13]. With this in mind, increased vaccination coverage due to COVID-19 vaccine mandates could reduce the health-related and economic impacts of the virus [13]. In addition, disease prevention among HCWs could reduce the risk of understaffing in healthcare facilities [14]. On the other hand, from a behavioral viewpoint, COVID-19 vaccine mandates could induce political polarization and further mistrust in vaccines [15]. In addition, divergences in global health policies, exclusion from work and social life, and depletion in healthcare system capacity may also occur due to COVID-19 vaccine mandates [15,16].

**Figure 1:**
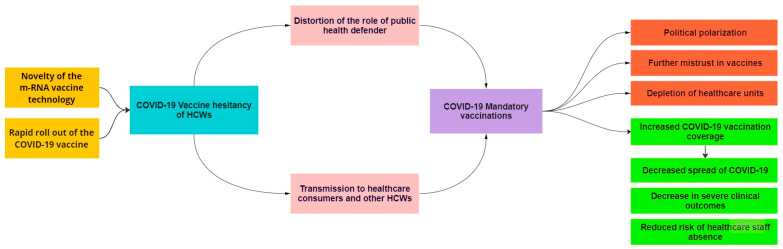
Conceptual framework: HCWs and COVID-19 vaccine mandates.

The aim of our study is to understand HCWs’ attitudes towards COVID-19 vaccine mandates, to assist policy makers in evidence-based decision making. We summarize the complex interactions between various determinants that play an important role in the chain of events concerning HCWs and their stance towards the COVID-19 vaccine mandates (Figure 1).

## 2. Materials and Methods

This systematic review and meta-analysis was reported according to the Preferred Reporting Items for Systematic Reviews and Meta-Analyses (PRISMA) [17].

### 2.1. Search Strategy and Eligibility Criteria

A systematic literature search was performed between July 2022 and November 2022 including articles from 5 databases: PubMed, Embase, Scopus, CINAHL, and Web of Science. In our search, only articles written in English were included. The following search strategy was used for all data bases: ((healthcare workers) OR (healthcare workforce) OR (healthcare personnel) OR (healthcare students) OR (nurse students) OR (medical students) OR (medicine students) OR (dental students) OR (dentistry students)) AND ((mandatory) OR (compulsory) OR (obligatory) OR (required)) AND ((COVID-19) OR (SARS-CoV-2)) AND ((vaccination) OR (immunization)). EndNote Web was used to import references from all databases and remove duplicates. After the removal of duplicates, the manual screening of abstracts/titles was performed by two independent researchers (M.P and G.R.), who applied the inclusion/exclusion criteria for each citation, and disagreements were resolved by consensus. During the abstract/title screening, 1597 articles were excluded as they were irrelevant to the research questions. A double screening strategy followed by a consensus (M.P and G.R.) was also adopted for the full text screening and the application of the inclusion/exclusion criteria to the screened studies. Last, all included studies were evaluated for risk of systematic bias by two independent researchers (M.P, G.R.) using Loney’s criteria, and disagreements were solved by consensus [18].

#### 2.1.1. Inclusion Criteria

1. Population: For this review, HCWs were defined as active professionals from all health-related professions (physicians, nurses, midwives, pharmacists, healthcare students, healthcare administration staff, etc.), with any vaccination status against COVID-19.
2. Study design: Original quantitative studies were included.
3. Outcomes: Articles that investigated the views and attitudes of HCWs towards mandatory COVID-19 vaccines of any type were included in this systematic review.

#### 2.1.2. Exclusion Criteria

1. Population: Non HCW populations were not eligible for this systematic review. In addition, studies on retired HCWs did not meet the eligibility criteria.
2. Study design: Studies of qualitative design were excluded.
3. Outcomes: Studies that did not analyze data on HCWs’ views and attitudes towards mandatory COVID-19 vaccines of any type were not included.

### 2.2. Data Extraction and Synthesis

Single-author data extraction (M.P.) was adopted, while a second author was responsible for quality checking of the extracted data (G.R.). The main themes used to determine which data were extracted were: 1. HCWs’ acceptance towards COVID-19 vaccination mandates for the general population, 2. HCWs’ acceptance towards COVID-19 vaccination mandates for HCWs. Moreover, in the third and last section of the study, we summarized broad evidence regarding the views and attitudes of HCWs towards mandatory COVID-19 vaccinations. Details of the studies, such as names of first authors, year of publication, sample size, and country, were extracted, along with various socio-demographic factors (age of participants, gender, profession, etc.). Tabulated data of the available information were used for comparison between the included studies. Sample size in meta-analyses may differ from those tabulated, as not all the responders answered the questions regarding COVID-19 mandates in each individual study. The total number of participants in the papers assessed in our study was 77,466 HCWs.

Narrative synthesis was used for combining evidence from all the included studies when the data were not compatible with meta-analysis, but also for summarizing the main findings of the meta-analyses, according to the Cochrane Consumers and Communication Review Group guidelines [19]. STATA 17 software was employed to perform meta-analyses using the metan routine and logit transformation of the proportions [20,21]. For the theme “HCWs’ acceptance towards COVID-19 vaccination mandates for HCWs”, 30 studies (n = 38,839 participants) were eligible for inclusion, while for the theme “HCWs’ acceptance towards COVID-19 vaccination mandates for the general population”, 22 studies (n = 24,882 participants) were included. Binary data on HCWs’ acceptance towards COVID-19 vaccine mandates were used in meta-analyses. Where binary data were not available, the data were dichotomized as follows: for a 3-point scale, agree = 0, while neutral and disagree = 1 [22], and for a 5-point scale, strongly agree and agree = 0 while neutral, disagree, and strongly disagree = 1 [22]. Only the random effects model was used in our analysis, as considerable heterogeneity between the individual studies was observed (I^2^ > 95%), which is common in meta-analyses of proportions [23]. The outcome of the meta-analyses was measured as a pooled proportion with corresponding 95% confidence intervals [23].

### 2.3. Sensitivity Analysis and Individual Study Risk of Bias Assessment

Sensitivity analysis was performed at two levels: 1. alternative dichotomization and 2. exclusion of the studies at high risk of systematic bias. Regarding alternative dichotomization, a random effects meta-analysis model was performed using the data as follows: for a 3-point scale, agree and neutral = 0, while disagree = 1. For a 5-point scale, strongly agree, agree, and neutral = 0, while disagree and strongly disagree = 1 [22,24].

A second sensitivity analysis was conducted based on the results of critical appraisal of the individual studies (Supplementary Materials). Loney’s critical appraisal tool for observational studies had the highest score of 8, taking into account the number of participants, the sampling method and frame, the credibility of the measurement tool, the participation rate and the descriptions of the refusers, as well as the characteristics of the participants and the provision of confidence intervals, along with the effect size [18]. We arbitrarily decided upon a cut-off point of <4/8 as a limit for exclusion during the risk of bias sensitivity analysis.

### 2.4. Sub-Group Analysis

Due to the high observed heterogeneity between the individual studies, a subgroup analysis was deemed appropriate. Three subgroup analyses were conducted by stratifying data by year of publication, W.H.O. region, and health profession (physicians and other health professions).

### 2.5. Publication Bias Risk Assessment

Many authors have discussed the need for a different approach to the funnel plot when it comes to publication bias risk assessment in meta-analyses of proportions [25,26]. With this in mind, we decided to evaluate the risk of publication bias using a DOI plot and the LFK index [27]. An LFK index > |1| suggests minor asymmetry, while an LFK index > |2| suggests major asymmetry in the distribution of individual effect sizes around the pooled effect size, assuming an increased risk of publication bias [27]. However, as funnel plots are still the most popular method for assessing publication bias, we also produced funnel plots, along with the corresponding Egger’s test for a more comprehensive assessment.

## 3. Results

**Figure 2:**
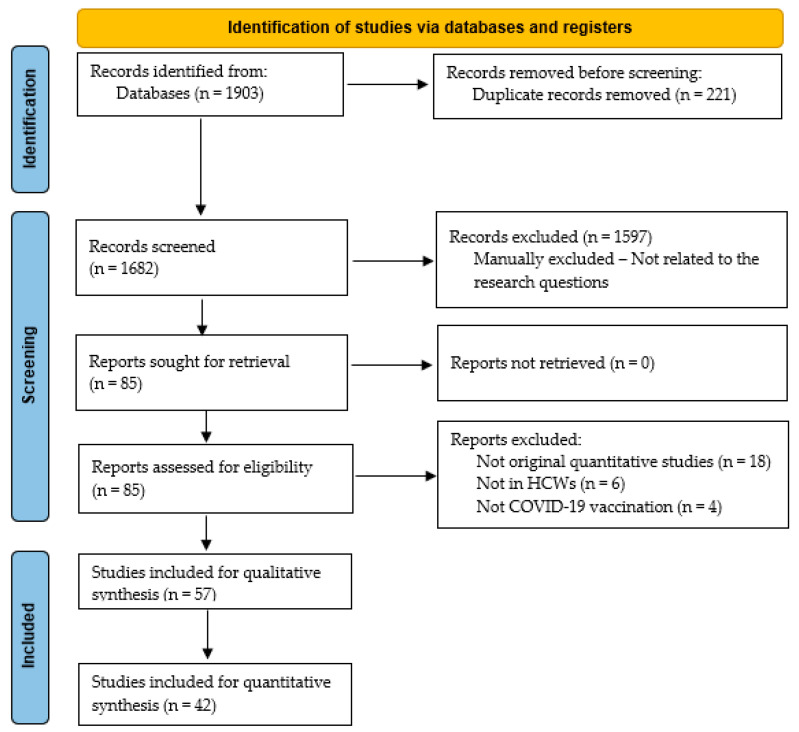
Flowchart of our systematic literature search (July 2022–November 2022).

After conducting systematic searches in PubMed, CINAHL, Web of Science, Scopus, and Embase, we retrieved 1903 articles. After duplicate removal (221 results), 1682 articles were retrieved for title and abstract screening. Our screening resulted in a collection of 85 articles. After applying the inclusion/exclusion criteria, we excluded 28 articles for specific reasons (Figure 2). Finally, 57 cross-sectional studies with 77,466 participants were included in this systematic review, while 42 were selected for the quantitative synthesis of evidence. The main findings of the 16 articles not considered for meta-analysis are discussed in the third section.

### 3.1. HCWs’ Acceptance of COVID-19 Vaccination Mandates for the General Population

#### 3.1.1. Main Findings

Regarding the acceptance of HCWs towards mandatory COVID-19 vaccination for the general population, 22 studies were considered for quantitative evidence synthesis. The total number of participants was 24,882 HCWs, coming from 13 different countries (Table 1). Considerable differences between the individual studies were observed (from 11% to 84% acceptance), while HCWs’ acceptance rate was ≥ 50% in 12 out of 22 studies. Four studies from Saudi Arabia were included; these showed a general trend of HCWs taking a supportive stance towards COVID-19 vaccine mandates for the general population, which was not the case for the single included study from Cyprus [28,29,30,31,32]. Three studies from Turkey produced conflicting results, while HCWs were clearly against COVID-19 vaccine mandates for the general population in two studies from France and the UK [33,34,35,36,37]. Additionally, evidence from an Austrian study suggests that HCWs were equally divided about accepting COVID-19 vaccine mandates for the general population [38]. About a third of HCWs were in favor of mandatory vaccinations against COVID-19 in a study from Slovenia, while in a study conducted in the Czech Republic, HCWs supported mandatory COVID-19 vaccines for the general population [39,40]. All three included studies from Italy resulted in similar findings, reporting that most of HCWs supported mandatory COVID-19 vaccinations for the general population [41,42,43]. Another four studies (three from the US and one from Barbados) investigated the acceptability of mandatory COVID-19 vaccinations for the general population [44,45,46,47]. In the aforementioned studies, the acceptance rate for mandatory COVID-19 vaccination ranged from 29% to 66%. Finally, respondents in the two included studies from Egypt and Sudan appeared to be divided about COVID-19 vaccine mandates for the general population, with acceptance rates of 50% and 54% [48,49].

#### 3.1.2. Meta-Analysis

Regarding the acceptance of HCWs towards COVID-19 vaccine mandates for the general population, 22 studies with 24,882 participants from 13 different countries were considered for quantitative evidence synthesis.

**Figure 3:**
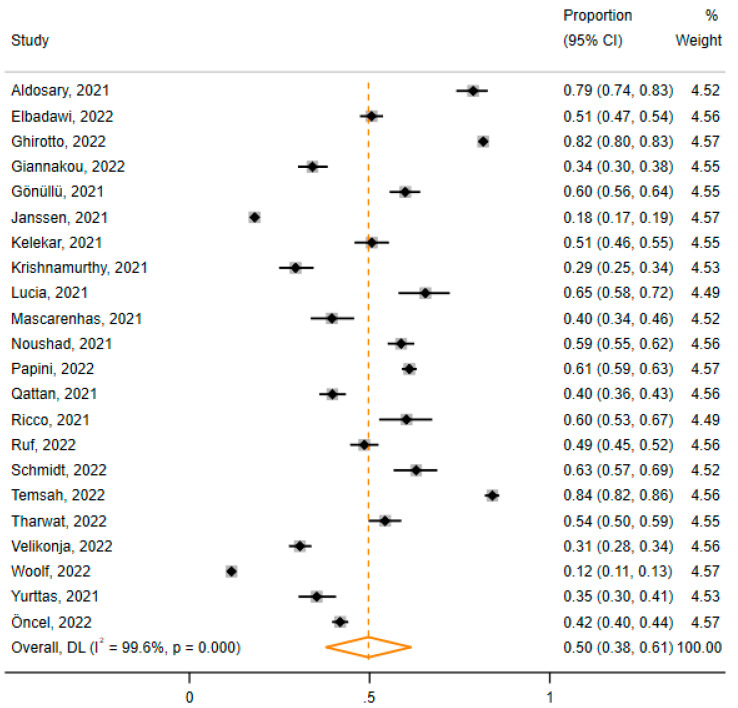
Results of the meta-analysis. The pooled proportion (orange rhombus) of HCWs’ acceptance of mandatory COVID-19 vaccines for the general population was 50% (95% CI: 38%, 61%) [28,29,30,31,32,33,34,35,36,37,38,39,40,41,42,43,44,45,46,47,48,49].

Using the random effects model due to the considerable observed heterogeneity between the included studies (I^2^ = 99.6%, Cochrane Q test *p*-value = 0.000), we calculated a pool estimate of 50% (95% CI: 38%, 61%) HCWs’ acceptance towards COVID-19 vaccine mandates for the general population (Figure 3).

**Figure 4:**
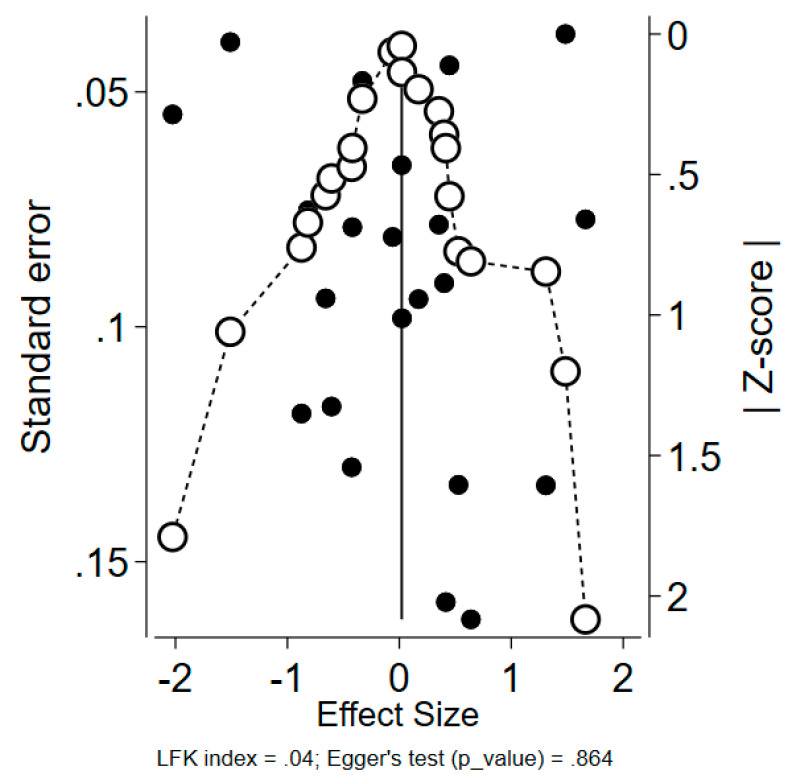
DOI plot with LFK index = 0.04, along with the funnel plot and Egger’s test (*p*-value = 0.864).

The publication bias risk assessment was conducted using both a DOI plot and a funnel plot. According to the LFK index, with a cut-off point of |2| (minor vs. major asymmetry), no asymmetry was observed, indicating that no risk of publication bias was detected (Figure 4). Considering all the limitations of using funnel plots in meta-analyses of proportions, we also did not identify any considerable visual asymmetry in alignment with the corresponding Egger’s test (*p*-value = 0.864).

### 3.2. HCWs’ Acceptance of COVID-19 Vaccination Mandates for HCWs

#### 3.2.1. Main Findings

A total of 30 studies with 38,839 participants were considered for meta-analysis regarding the outcome “acceptance of HCWs towards mandatory COVID-19 vaccination for HCWs” (Table 2). Despite the wide evidence heterogeneity, a general trend of support towards COVID-19 vaccine mandates was observed. In 21 of the 30 studies, HCWs placed themselves in favor (>50% acceptance) of mandatory COVID-19 vaccinations for HCWs. With regard to Europe, four studies from Italy were included, which presented a generally supportive stance towards COVID-19 vaccination mandates for HCWs [43,50,51,52]. All three included studies from Greece consistently showed that HCWs had supportive attitudes towards COVID-19 vaccine mandates for HCWs, unlike the two studies from France, whose authors reported a general trend of opposition [36,53,54,55,56]. In the single included study from Cyprus, HCWs were divided on their attitudes towards COVID-19 vaccine mandates [32]. The last three studies from Europe (UK, Poland, and the Czech Republic) showed conflicting results regarding HCWs’ acceptance of vaccine mandates for HCWs (acceptance rates: 6–75%) [37,40,57]. A generally supportive attitude was reported in a series of seven studies from the US, while data from two Australian studies revealed that Australian HCWs were divided about accepting COVID-19 vaccine mandates for HCWs [44,45,46,58,59,60,61,62,63]. In three studies (one from India, one from Mongolia, and one from Pakistan), most participants were in favor of vaccine mandates for COVID-19, while in another study from India, 60% of participants were against them [64,65,66,67]. Additionally, in a study conducted in three Asian countries (Hong Kong, Vietnam, and Nepal), participants were almost equally divided regarding their attitudes towards COVID-19 vaccine mandates for HCWs [68]. Last, three more studies (Tanzania, Sudan, and Egypt) were included, resulting in conflicting results, as two of them suggested a strong supportive attitude towards vaccine mandates for COVID-19, while Konje et al. reported a 30% acceptance rate [48,49,69].

#### 3.2.2. Meta-Analysis

A total of 30 studies with 38,839 participants from 18 different countries were considered for the quantitative synthesis of evidence regarding the outcome “HCWs’ acceptance of COVID-19 vaccination mandates for HCWs”.

**Figure 5:**
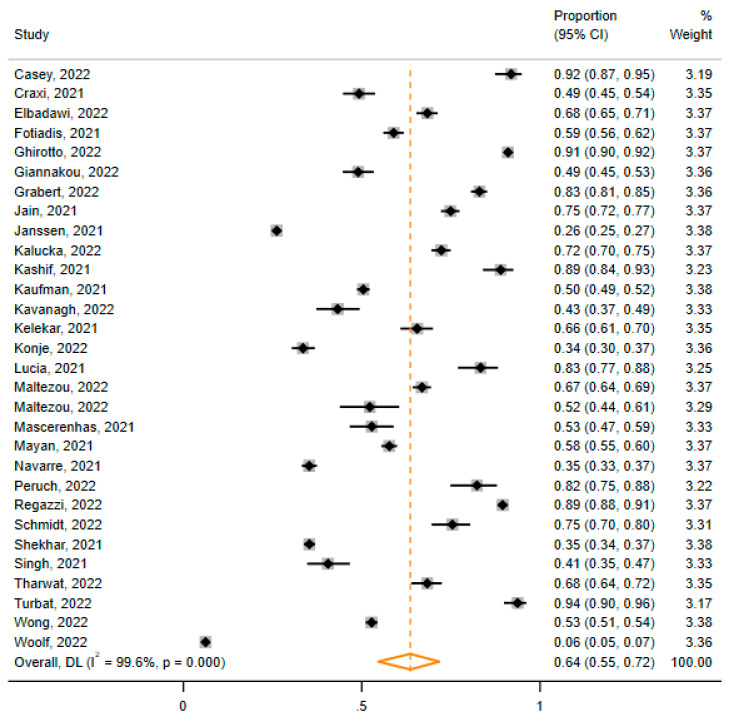
Results of the meta-analysis. The pooled proportion (orange rhombus) of HCWs’ acceptance of mandatory COVID-19 vaccines for HCWs was 64% (95% CI: 55%, 72%) [32,36,37,40,43,44,45,46,48,49,50,51,52,53,54,55,56,57,58,59,60,61,62,63,64,65,66,67,68,69].

Again, due to the observed heterogeneity between the individual studies, only the random effects model was performed (I^2^ = 99.6%, Cochrane Q test *p*-value = 0.000). The estimated pooled proportion was 64% (95% CI: 55%, 72%) (Figure 5).

**Figure 6:**
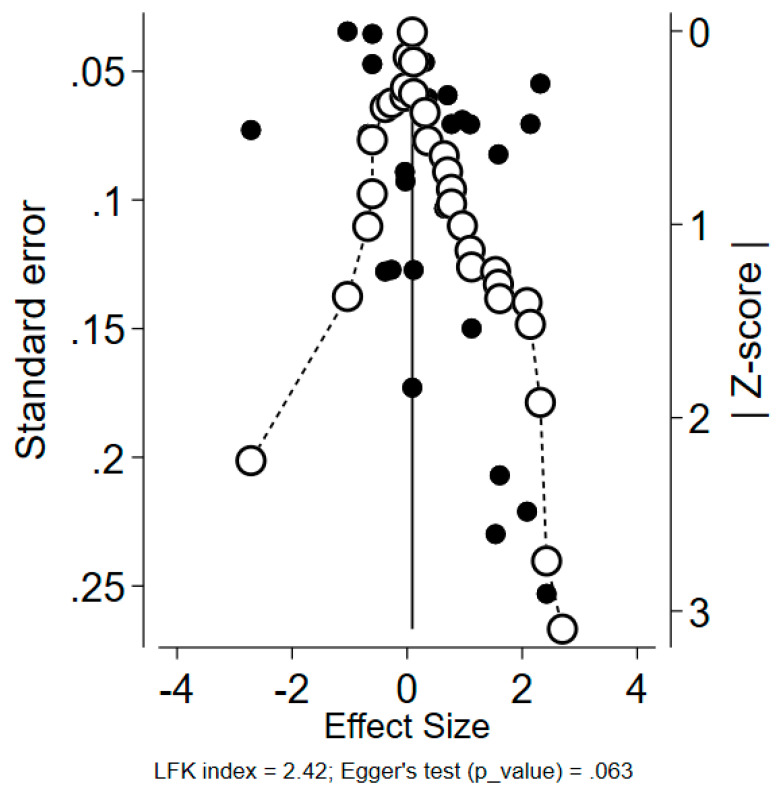
DOI plot with LFK index = 2.42, along with the funnel plot and Egger’s test (*p*-value = 0.063).

The publication bias risk was assessed using a DOI plot and the corresponding LFK index, along with a funnel plot and Egger’s test. As can be seen below, major positive asymmetry is observed (LFK index = 2.42), indicating a high risk of publication bias (Figure 6). Even if Egger’s test significance is on the borderline, visual asymmetry in the funnel plot can be seen, as well. Studies with the highest standard error reporting extreme rates of COVID-19 vaccine mandate acceptance (>80%) are probably overrepresented in the current meta-analysis.

### 3.3. Sub-Group and Sensitivity Analysis

#### 3.3.1. Sensitivity Analysis and Comparison of the Meta-Analysis Results

A comparison between the primary analysis results and the results of the sensitivity analyses was conducted to control for the effect of data dichotomization, as well as for the risk of systematic bias in the individual studies (Table 3). The general trend of higher acceptance (10–14%) from HCWs towards COVID-19 vaccine mandates for HCWs remained consistent after analyzing the data using all three approaches.

Alternative dichotomization sensitivity analysis resulted in a pooled estimate of 55% for HCW acceptance of mandatory COVID-19 vaccines for the general population, while for HCWs, the corresponding acceptance rate was 67%. Regarding publication bias risk assessment during the alternative dichotomization sensitivity analysis, for the outcome “HCWs’ acceptance of COVID-19 vaccination mandates for HCWs”, we identified major asymmetry using both the DOI and funnel plots (LFK index = 3.06, Egger’s test *p*-value = 0.028), whereas for the outcome “HCWs’ acceptance of COVID-19 vaccination mandates for the general population”, no asymmetry was observed (LFK index = 0.00, Egger’s test *p*-value = 0.528).

A second sensitivity analysis was carried out that excluded studies at high the risk of systematic bias. According to the risk of bias sensitivity analysis, HCWs’ COVID-19 vaccine mandates acceptance rates were 45% for the general population and 55% for HCWs. As for the publication bias risk assessment in the risk of bias sensitivity analysis, minor asymmetry was observed using both the DOI and funnel plots (LFK index = 1.31, Egger’s test *p*-value = 0.135) regarding HCWs’ acceptance towards COVID-19 vaccine mandates for HCWs; meanwhile, no asymmetry was observed regarding HCWs’ acceptance of mandatory COVID-19 vaccination for the general population (LFK index = 0.49, Egger’s test *p*-value = 0.197).

#### 3.3.2. Sub-Group Analysis

In order to investigate potential factors contributing to the significant observed heterogeneity, three subgroup analyses were performed:

##### Sub-Group Analysis by W.H.O. Region

A sub-group analysis was performed by stratifying the data according to W.H.O. region (Table 4). No statistically significant subgroup effect was identified (*p*-value = 0.712, *p*-value = 0.208), while the within-subgroup heterogeneity remained considerably high (I^2^ > 95%, Cochrane Q test *p*-value = 0.000).

##### By Year of Publication

A second sub-group analysis was performed based on the year of publication of the individual studies. Although an upward trend in the acceptance of HCWs towards COVID-19 vaccine mandates was observed for both outcomes between 2021 and 2022 (13% for HCWs, 3% for the general population), publication year did not have a significant subgroup effect (*p*-value = 0.148, 0.822), while within-subgroup heterogeneity remained high in all sub-groups (l^2^ > 95%, Cochrane Q test *p*-value = 0.000) (Table 4).

##### By Occupational Status (Physicians and Other HCWs)

**Figure 7:**
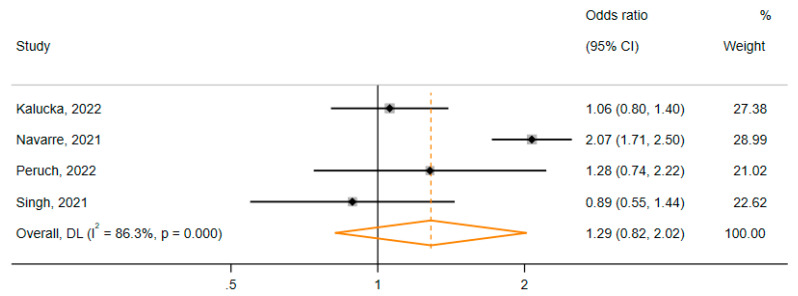
Relationship between physicians and other HCWs regarding the acceptance of COVID-19 vaccine mandates for HCWs [52,56,57,65].

In our third approach to subgroup analysis, we sought to explore the effect of occupational status on acceptance of COVID-19 vaccine mandates. Only four studies reported stratified data in relation to the outcome “HCWs’ acceptance of COVID-19 vaccine mandates for HCWs”. Thus, only one subgroup analysis was performed, yielding an odds ratio of 1.29 (95% CI: 0.82, 2.02) (Figure 7). Although physicians appeared to have 29% increased odds of accepting the COVID-19 vaccine for HCWs compared to other HCWs, this relationship was insignificant in our model.

### 3.4. Main Findings of the Studies Not Included in Quantitative Synthesis of Evidence

In the last section, we include studies that were not considered for meta-analysis due to a lack of compatible data. Therefore, only a narrative synthesis of evidence was conducted. The demographic features and main findings of these studies are tabulated in Table 5 and Table 6. In total, 16 studies (n = 28,560 participants) from 13 different countries were considered for narrative synthesis of evidence.

#### 3.4.1. COVID-19 Vaccine Mandates as a Working Requirement

Four studies addressed the attitudes of HCWs towards mandatory COVID-19 vaccination as a working requirement. In a large US study, 90.5% of HCWs decided to be vaccinated after a COVID-19 vaccine working requirement was announced, while 26.7% remained unvaccinated when the COVID-19 vaccine was not a working requirement [70]. Furthermore, a study from Nigeria reported that 52.3% of HCWs would be vaccinated if it was imposed by the institutional heads, while 95.8% of HCWs in a study from Mongolia agreed with the COVID-19 vaccine mandates as a working requirement [64,71]. Last, Poyiadji et al. reported that most of the HCWs of a US radiology department complied with the COVID-19 vaccine mandates, resulting in almost no disruption in the operation of the department [72].

#### 3.4.2. COVID-19 Vaccine Mandates for Hesitant HCWs

Regarding HCWs’ COVID-19 vaccine acceptance, mandatory COVID-19 vaccinations did not seem to be an adequate factor in opinion change, as reported by a study from Jordan [73]. In alignment with this, a study from Saudi Arabia reported that vaccine mandates decreased the odds of vaccine acceptance by 27% [74]. Similarly, as reported in a Chinese study [75], only 2% of participants would change their decision to not be vaccinated due to the imposition of COVID-19 vaccine mandates. In a Swiss study 14% of the participants reported that COVID-19 mandatory vaccination could be an important factor in changing attitude about COVID-19 vaccination only for certain reasons (e.g., travel) [76]. On the other hand, Costantino and Masood reported that COVID-19 vaccine mandates were an important factor for in hesitant HCWs changing their opinion regarding the COVID-19 vaccination [77,78].

#### 3.4.3. COVID-19 Vaccine Mandate Acceptance/Agreement

In the single included study from Poland, most of the participants agreed that the COVID-19 vaccine should be mandatory for HCWs (median: 4, Q3–Q5), in alignment with the findings of Ciliberti et al., where Italian healthcare students supported COVID-19 vaccine mandates for the whole community and for students [79,80]. In a study from Greece, it was seen that HCWs who supported mandatory COVID-19 vaccinations were more prone to becoming vaccinated than those who were opposed to them (84% and 19%, respectively) [81]. Ulbrichtova et al. reported that profession (being a physician) or/and vaccination status (being vaccinated) were important factors in the acceptance of vaccine mandates [82]. Furthermore, in a study with Arab-speaking HCWs from around the world, only 16.2% of the participants supported COVID-19 vaccine mandates for specific groups of people, e.g., for those for whom the COVID-19 vaccine has been proven to be safe and effective according to clinical trials [83]. Last, evidence from a study conducted in Oman suggest that male and older HCWs had a more supportive stance towards COVID-19 vaccine mandates when compared to their female and younger counterparts [84].

**Table 5:** Demographic features of the studies not included in quantitative synthesis of evidence.

Study	Country	Participants	Gender (Female)	Age (Years)	Profession
[73]	Jordan	n = 287	190 (66.2%)	mean: 26.8 ± 8.9 (SD)	Medical field workers
[74]	Saudi Arabia	n = 529	362 (68%)	-	Physicians: 88 (16.64%) Nurses: 223 (42.16%) Administrators: 41 (7.75%) Allied health professionals: 23 (4.35%) EMS: 1 (0.19%) Pharmacists: 16 (3.02%) Technicians: 28 (5.29%) Other: 109 (20.60%)
[84]	Oman	n = 346	156 (45%)	Male, mean age ± SD: 46.8 ± 9.2 Female, mean age ± SD: 40.3 ± 7.6	Physicians: 165 (47.7%) Nurses: 181 (52.3%)
[80]	Italy	n = 244	Female: 163 (68.2%) Male: 70 (29.3%) No answer: 6 (2.5%)	22.3 years (range 19–35)	Medical Students
[77]	Italy	n = 1450	939 (64.7%)	Mean age: 46.3 ± 15.7 (SD)	Pharmacists: 1450 (100%)
[70]	USA	n = 12,875	9358 (73%)	18–29: 2305 (18%) 30–49: 5750 (45%) 50–64: 3744 (29%) 65+: 919 (8%)	Healthcare personnel
[79]	Poland	n = 497	-	median (Q1–Q3) age was 24 (21–28)	Non-medical staff: 8 (1.6%) Other medical staff: 14 (2.8%) Students: 333 (67.1%) Nurses: 108 (21.7%) Midwives: 19 (3.8%) Paramedics: 2 (0.4%) Doctors: 13 (2.6%)
[81]	Greece	n = 1591	1004 (63%)	< 30: 282 (17.7%) 31–40: 363 (22.8%) 41–50: 450 (28.3%) > 50: 496 (31.2%)	Physicians: 480 (31.6%) Nursing personnel: 607 (39.9%) Paramedical personnel: 171 (11.2%) Supportive personnel: 72 (4.7%), Administrative personnel: 191 (12.6%)
[78]	Pakistan	n = 331	175 (53%)	<30: 183 (55%) 30–40: 93 (28%) 41–50: 26 (8%) 50–60: 22 (7%) >60: 7 (2%)	Physicians: 94 (28%) Nurse/nursing assistants: 95 (29%) Technologists/technicians: 118 (36%) Medical social officers: 24 (7%)
[71]	Nigeria	n = 440	224 (50.9%)	<25: 296 (67.3%) >25: 144 (32.7%)	Doctors of medicine: 166 (37.7%) Pharmacy staff: 133 (30.2%) Nursing staff: 103 (23.4%) Others: 38 (8.6%)
[72]	USA	n = 1506	77.1%	44.1 ± 13.3	Radiology department employees: 1506 (100%)
[76]	Switzerland	n = 776	Female: 651 (84%) Male: 102 (13%) Missing information: 23 (3%)	-	Nurses: 332 (43%) Auxiliary nursing staff: 34 (4%) Patient care technicians: 4 (1%) Administration staff: 95 (12%) Respiratory, physical, or speech therapist: 55 (7%) Social workers: 2 (0%) Other: 24 (3%) Missing information: 22 (3%)
[83]	Arab Countries	n = 5708	2537 (44.4%)	30.6 years (±10)	HCWs
[64]	Mongolia	n = 238	195 (81.9%)	18–25: 18 (7.6%) 26–35: 148 (62.2%) 36–45: 48 (20.2%) 46–55: 20 (8.4%) >55: 4 (1.7%)	Physicians: 162 (68.1%) Other: 76 (31.9%)
[82]	Slovakia	n = 1124	997 (78.1%)	mean age: 48.3 ± 12.6 (SD)	Physicians: 582 (52%) Non-physician HCWs: 542 (48%)
[75]	China	n = 618	581	-	Physicians: 322 (55%) Nurses/midwives: 235 (40%) Laboratory staff and others: 61 (5%)

**Table 6:** Main findings of the studies not included in quantitative synthesis of evidence.

Study	Country	Main Findings
[73]	Jordan	Factors affecting the willingness to be vaccinated for COVID-19; 25.4% answered: mandatory in schools, universities, and workplaces
[74]	Saudi Arabia	Vaccine mandates decreased the OR of vaccine acceptance by 27%
[84]	Oman	Male and older HCWS had a more supportive stance towards mandatory COVID-19 vaccination when compared to their female and younger counterparts
[80]	Italy	Healthcare students believed that COVID-19 vaccine mandates should be compulsory for the whole population, including students. Furthermore, the participants stated that students who refuse COVID-19 vaccination should be excluded from university (8–10 (Likert-type answers medians))
[77]	Italy	A total of 64.3% of those who changed their opinion regarding COVID-19 vaccination did so due to vaccines mandates
[79]	Poland	Most of the participants agreed that COVID-19 vaccines should be mandatory for HCWs (median: 4, Q3–5)
[81]	Greece	HCWs who supported COVID-19 vaccine mandates for HCWs were more prone to being vaccinated (83.9%) against COVID-19 when compared to those who did not support COVID-19 vaccine mandates (19%) for HCWs
[78]	Pakistan	A total of 59% of the participants answered that official requirements were their reason for being vaccinated
[72]	USA	The majority of HCWs showed compliance with vaccine mandates. Almost no disruption in the operation capacity of healthcare settings was shown
[76]	Switzerland	Reasons that may change participants’ minds regarding COVID-19 vaccination: mandatory vaccination for certain situations (e.g., travel) (11/79 (14%))
[70]	USA	A total of 90.5% of HCWs who faced working requirements had been vaccinated against COVID-19, as compared to 73.3% of HCWs without vaccination requirements (24% increased odds)
[71]	Nigeria	A total of 52.3% would undergo COVID-19 vaccination if mandated by the heads of institution
[83]	Arab-speaking HCWs	Only 16.2% of HCWs supported mandating the vaccine in some groups of people
[64]	Mongolia	A total of 95.8% of HCWs agreed with vaccine mandates as a working requirement
[82]	Slovakia	Profession (being a physician) or/and vaccination status (being vaccinated) were important factors in the acceptance of vaccine mandates
[75]	China	A total of 2% of vaccinated HCWs were unwilling to be vaccinated but followed the employers’ mandates

## 4. Discussion

### 4.1. Origins of Controversy Regarding COVID-19 Vaccine Mandates

To our knowledge, this is the first published systematic review and meta-analysis that investigates the attitudes of HCWs towards COVID-19 vaccine mandates for the general population and HCWs. Our results indicate that COVID-19 vaccine mandates are a highly controversial topic among healthcare professionals. HCWs were divided regarding the imposition of COVID-19 vaccine mandates on the general population, while only a weak majority of them supported the imposition of COVID-19 vaccination on HCWs. Regarding the causes of the controversy, Rodger et al. highlighted that concerns about the safety and effectiveness of the COVID-19 vaccine, as well as distrust in the pharmaceutical industry and governmental/public health officials, were crucial factors in the opposition to COVID-19 vaccine mandates [85]. Other authors additionally mentioned that a proportion of HCWs believed that COVID-19 vaccination was unnecessary, expressing the opinion that natural immunity was preferable to vaccination and that previously infected people should not be vaccinated [7,86]. Moreover, older age and prior COVID-19 infection seemed to be important factors in COVID-19 vaccine acceptance, as younger HCWs and those who had not yet been infected were more hesitant to receive the COVID-19 vaccine [86]. The latter might reflect the experienced severity of COVID-19 infection and the differences in risk perception between younger and older HCWs, as older age is the most important risk factor in severe COVID-19 [87]. Numerous studies suggest a positive relationship between vaccination status and/or the intention of being vaccinated and the acceptance of COVID-19 vaccine mandates [28,32,38,44,48,66,81,82,88]. With this in mind, it is not arbitrary to assume that the HCWs who were more prone to supporting mandatory COVID-19 vaccinations were those at higher risk of infection or a severe clinical outcome (frontline HCWs and older HCWs). Moreover, HCWs who had undergone other compulsory vaccinations in the past were more prone to being vaccinated with the COVID-19 vaccine, underlining the importance of vaccine literacy among HCWs [88].

As already mentioned, safety concerns were prominent among HCWs, and although the benefits of COVID-19 vaccination clearly outweigh the risks, the latter are worth mentioning. Many countries decided to suspend the Oxford/AstraZeneca and the Johnson & Johnson vaccines due to a small risk of blood clots, while the mRNA vaccines were related with mild cases of myocarditis [14]. The argument that HCWs were opposed to COVID-19 vaccine mandates due to safety reasons takes on greater strength when one considers that even for the long-tested flu vaccine, HCWs’ oppositions were based on concerns about the acute side effects or middle- and long-term effects (Guillain–Barré syndrome and thimerosal exposure) [89].

### 4.2. HCWs’ Moral Imperatives Regarding Vaccinations

Vaccine mandates are at the center of interest when it comes to limitations to individual liberties, but this becomes more complex when we consider HCWs’ ethical and professional obligation to minimize the risk of harming their patients [14]. Indeed, vaccinations are a well-established medical procedure that contributes both to illness and transmission prevention [14]. However, regardless their proven benefits, vaccinations are not often a legal requirement for HCWs, but rather, a part of the Good Medical Practice code or an obligation towards the professional bodies [14]. In our view, the estimated 14% difference in HCWs’ acceptance of COVID-19 vaccine mandates between the general population and HCWs largely embodies the moral obligation of HCWs not to harm their patients. Of course, a considerably large proportion of HCWs do not agree with the COVID-19 vaccine mandates both for the general population and HCWs. Decision and policy makers should be aware of this important proportion of HCWs who are opposed to vaccine mandates. This is of particular importance, as vaccination mandates have been found to be associated with the aggravation of distrust in officials, the depletion of healthcare facilities, political polarization, and decreased intent to receive both the COVID-19 vaccine and unrelated vaccines, such as the chickenpox vaccine [15,90].

### 4.3. The Relationship between COVID-19 Vaccination Status and/or Intention to Vaccinate against COVID-19 and Acceptance of COVID-19 Vaccine Mandates

Wide global differences have also been seen in healthcare workers’ willingness to be vaccinated with the COVID-19 vaccine, as revealed in a review of 26 studies [91]. According to this review, the willingness of HCWs to be vaccinated with the COVID-19 vaccine ranged from 36% to 82.95%, while in two more recent studies, this trend remained stable, with the reported results being 70.5% and 40%, respectively [48,92]. Being vaccinated or having a strong intention to be vaccinated with the COVID-19 vaccine is followed by HCWs’ support towards COVID-19 vaccine mandates [28,32,38,44,48,66,81,82,88]. The close relationship between HCWs’ willingness to vaccinate against COVID-19 and their supportive attitudes toward COVID-19 vaccine mandates is particularly important, given that factors influencing HCWs’ intentions to vaccinate have been extensively studied. With this in mind, these factors should be taken into consideration when mandatory vaccination policies are formed. According to Willems et al., attitudes related to vaccination willingness are related to the threat of COVID-19, responsibility towards the community, vaccine mandates, the protection of family members, and the vaccination as a working requirement [91]. More evidence is needed that explores the relationship between HCWs’ willingness to be vaccinated and/or vaccination status, and their support for mandatory vaccinations. The use of such evidence is expected to increase vaccination-related policy implementation rates, especially during emerging epidemics/pandemics.

### 4.4. Other Vaccine Mandates

Other authors have attempted to assess HCWs’ acceptance of mandatory vaccinations for diseases other than COVID-19. In a meta-analysis, the pooled proportion of HCWs favoring mandatory influenza vaccinations was 61% (95% CI: 53%, 68%) [93]. Although no distinction was made regarding attitude orientation (for HCWs or for the general population), this previous study reports results similar to ours. These results prioritize the restriction of individual liberties as the primary reason for HCWs’ opposition to vaccine mandates, as the influenza vaccine is known to be an effective, safe, and widely used vaccine, yet universal acceptance by health professionals was not observed [94]. This argument is consistent with the results reported in a Greek study where acceptance was the second highest for COVID-19 vaccine mandates among eight vaccines, slightly behind the hepatitis B vaccine [55]. Along the same lines, many HCWs were divided between a shared decision and vaccine mandates in a study conducted in Switzerland on MMR and influenza vaccines [95].

On the other hand, findings from an Australian study conducted in 2009 showed that the vast majority (78%) of health officials supported a new mandatory vaccination policy against certain infectious diseases (measles, mumps, rubella, diphtheria, tetanus, pertussis, hepatitis B, and varicella) for HCW, while only 3.6% were against it [96]. This also leads us to consider that the rapid release of the vaccine for COVID-19 and the innovation of mRNA technology may play an important role in the opposition to mandatory vaccination. Furthermore, while the COVID-19 vaccine mandates have led to mixed results in terms of vaccination coverage of HCWs, other mandatory vaccination campaigns have achieved coverage rates above 90%, as described in two systematic reviews regarding an influenza and Tdap mandatory vaccination campaign [13,70,71,72,73,91,97,98].

### 4.5. The Role of Top-Down Policies and Public Engagement

According to Buse et al., central decision makers, such as governmental officials and policy makers, have prominent roles in top-down implementation policy strategies, leaving not much room for other actors, such as regional authorities, the private sector, and frontline workers, to be engaged in decisions that have an important impact on them [99]. The current systematic review includes many studies from countries with a medium or low rate of public engagement and political participation, which may reflect these countries’ tendency for top-down policies [100,101]. On the contrary, governments of countries with a high rate of political participation, such as the Netherlands, called for public participation in policy decision making in the COVID-19 pandemic [102]. As previously discussed, HCWs’ hesitancy about the COVID-19 vaccine was an important factor that led governments to impose vaccination policies against COVID-19 [7,8,9,10,11,12]. Furthermore, different approaches to policy making, along with the impact of COVID-19 in each country, to some extent, explain the country composition of our systematic review [100,101,103,104,105]. According to the Inter-Parliamentary Union and the Organization for Economic Co-operation and Development (OECD), public engagement plays a critical role in addressing public distrust in governments, which has been one of the main reasons of COVID-19 vaccine hesitancy [85,106,107]. Furthermore, public engagement could be mutually beneficial for policymakers and the public, as members of parliaments could receive information that could improve proposed policies and their implementation, while citizens could see their different perspectives and opinions included in their countries’ policies [106,107]. Additionally, it should be noted that obligatory vaccinations are policies that reduce individual autonomy, and they should be justified by promoting and protecting public health [1]. In this context it should be also underlined that SARS-CoV-2 presents fundamental differences in comparison, e.g., to measles [108]. These differences include the phenotypic instability of SARS-CoV-2 and its current inability to produce long term protective immunity; therefore, it has been suggested that herd immunity may not be applicable to COVID-19 [108]. With respect to classical vaccine-preventable occupational hazards such as Hepatitis B Virus Infection (HBV), substantial heterogeneity related to vaccination policies for HCWs has been reported [109]. In that case, even an established vaccine intervention in terms of its safety and efficacy did not achieve universal mandatory implementation in contrast to COVID-19 vaccine.

### 4.6. Societal Impacts of COVID-19 Vaccine Mandates

By many authors, it has been proposed that vaccine mandates during the COVID-19 pandemic may threaten social cohesion by cultivating social polarization [15,110]. Mandatory vaccination of the general population and HCWs was not homogeneously adopted by states in the European Region [52]. For instance, France, Germany, Poland, Latvia, and Hungary did not adopt compulsory COVID-19 vaccination for the general population [52]. With respect to HCWs, several European countries (e.g., Italy, Germany, Greece, France, and Poland) implemented compulsory COVID-19 vaccination, while others (e.g., the Czech Republic and the United Kingdom) did not implement compulsory vaccination for HCWs [52]. These within- or between-country discrepancies in vaccination policy making could be a major threat to social cohesion and induce mistrustfulness towards current or future vaccination policies [15]. Although increased vaccination coverage against COVID-19 may limit the spread of the disease and reduce the risk of severe clinical outcomes, Bardosh et al. generally questioned the effectiveness of mandatory vaccination policies and highlighted the negative consequences of such policies [13,15].

### 4.7. Novel Scientific Evidence during the COVID-19 Pandemic

A very important finding is that our review is possibly biased by small studies that reported extremely high rates (>80%) of HCWs’ acceptance of COVID-19 vaccine mandates for HCWs. All of these studies were classified as being at high risk of systematic bias, and therefore, the magnitude of publication bias was thoroughly assessed during the risk of bias sensitivity analysis [111,112]. Poor methodological quality of small studies is a common cause of asymmetry both in DOI and funnel plots, suggesting the existence of publication bias [27,113]. It is well known that many editors are inclined towards publishing significant, positive, or even intriguing results, as these studies tend to be more citable [114,115]. The scientific community and policy makers should be aware of this and always think critically when interpreting such results. Above all, in emergencies such as the COVID-19 pandemic, new scientific evidence is selectively used to inform policy decisions, while also being part of polarized political debates [116]. Under urgent conditions, scientific competitiveness is the perfect fertile ground for such practices to flourish [116]. It is the responsibility of the scientific community to protect scientific evidence from politicization by prioritizing collaboration over competition.

### 4.8. HCWs’ Attitude Changes during the COVID-19 Pandemic

Evidence from a study conducted in Italy revealed that university students’ attitudes about COVID-19 vaccine mandates changed during the pandemic following pandemic trends [117]. Although we did not detect a statistically significant difference, HCWs’ views changed by 13% according to our sub-group analysis by year of publication. More research is needed on the specific factors influencing HCWs’ attitudes toward COVID-19 vaccine mandates during the ongoing pandemic. Timing is of great importance when it comes to public health policy making and should be taken seriously to achieve high rates of policy implementation [118].

### 4.9. Occupational Status and Acceptance of Vaccine Mandates

The relationship between professional status and the acceptance of mandatory vaccinations has been the subject of other scientific articles [93]. Regarding the acceptance of mandatory influenza vaccination, physicians appear to be more willing to receive a mandatory vaccine than nurses [93]. Although our results are based on only four studies, we reported a similar but insignificant trend for mandatory vaccination against COVID-19. The relationship between occupational status and the acceptance of mandatory vaccinations is something that should be thoroughly investigated in future studies.

### 4.10. Strengths and Limitations

Our study has several strengths and limitations. First, our study greatly contributes to the current literature by providing novel evidence of healthcare workers’ attitudes towards mandatory COVID-19 vaccinations for both HCWs and the general population. We identified and reported a high risk of publication bias in our meta-analysis. However, through our thorough sensitivity analysis, we assessed both the risk of systematic bias in the individual studies and the publication bias, providing three levels of evidence. Last, we included 57 studies with more than 75,000 participants, which significantly contributes to the reliability of our results.

However, our study has several limitations. First, as with observational studies, great heterogeneity was observed among the included studies [23,119]. This should be taken into account when one intends to generalize the results in a different country setting. The use of the random effects model due to high heterogeneity among the individual studies allowed us to report results with large 95% confidence intervals. We could not identify any cause of heterogeneity, despite the subgroup analysis. The heterogeneity may be due to the different designs of the studies, different features of the participants, different policies between countries, or time difference in the pandemic phase in which the studies were conducted. Furthermore, the cross-sectional design of the individual studies led us to provide results that incorporated evidence from different stages of the COVID-19 pandemic. More importantly, after the risk of bias assessment analysis, almost half of the studies included in our initial analysis were classified as being in the high risk of bias group. Last, since no data were available on HCWs’ acceptance of specific vaccine types (mRNA, etc.), our results include HCWs’ views on all vaccine types although there are indications that the introduction of new vaccine technologies plays an important role in vaccine hesitancy. However, in the vast majority of compulsory COVID-19 vaccination policies, there was freedom to choose between different types of vaccine.

## 5. Conclusions

### 5.1. Policy Implications and Recommendations

- Overall, 50% of HCWs opposed mandatory vaccination of the general population, and 36% of them opposed vaccine mandates for HCWs. These figures indicate that the mandatory COVID-19 vaccination of HCWs and the general population is a highly controversial topic among HCWs. In line with the W.H.O.s’ recommendations, our findings suggest that policy makers should prioritize other alternatives (e.g., information campaigns) over mandatory vaccination policies.
- We recognize the well-intentioned efforts of researchers to contribute to the scientific literature during emerging situations. In this context, policymakers should think critically before translating new evidence into policies, as our findings show that low-quality evidence with intriguing results tends to be published.

### 5.2. Suggestions for Future Research

- Further research is needed on the roles of different healthcare professionals regarding acceptance towards COVID-19 vaccine mandates.
- Understanding the relationship between vaccination status and/or intention to be vaccinated and the acceptance of vaccine mandates is expected to provide useful evidence for future vaccination policies.
- Our findings indicate that HCWs’ attitudes towards COVID-19 mandates may have changed during the pandemic. Determining the reasons for this change will inform policy makers as to the appropriate time of decision making.
- Important societal benefits are expected to arise as a result of clarifying the complex interactions between the different types of policy implementation and the role of the public engagement in the decision-making process during an epidemic/pandemic.

The present study provides stakeholders and policy makers with useful evidence related to the compulsory or non-compulsory nature of COVID-19 vaccination policy among HCWs and the general population. Moreover, the present findings may also inform vaccination policies in future pandemics.

## Figures and Tables

**Table 1:** Demographic features of the participants included in the meta-analysis: HCWs’ acceptance of COVID-19 vaccination mandates for the general population.

Study	Country	Participants	Gender (Female)	Age (Years)	Profession
[31]	Saudi Arabia	n = 334	-	-	HCWs
[49]	Sudan	n = 930	626 (67.3%)	Mean age: 28.7 ± 6.7 (SD)	Anesthesiologist: 2 (0.2%) Dentist: 92 (9.9%) Doctor: 585 (62.9%) Medical laboratory: 70 (7.5%) Nurse: 27 (2.9%) Pharmacist: 118 (12.7%) Physiotherapist: 4 (0.4%) Radiologist: 6 (0.6%) Staff in medical university: 26 (2.8%)
[43]	Italy	n = 4677	Female: 3132 (67.0%) Male: 1475 (31.5%) Not specified: 65 (1.4%) Other: 5 (0.1%)	18–30: 389 (8.3) 31–40: 866 (18.5) 41–50: 1169 (25.0) 51–60: 1380 (29.5) >60: 873 (18.7)	Health professionals: 2474 (52.9%) Nursing health professionals: 1230 (26.3%) Obstetric health professionals: 79 (1.7%) Rehabilitation health professionals: 482 (10.3%) Technical health professionals: 179 (3.8%) Prevention health professionals: 29 (0.6%) Health operators: 204 (4.4%)
[32]	Cyprus	n = 504	320 (63%)	Mean age: 36.7 ± 9.6 (SD)	Physicians: 62 (13.3%) Nursing staff: 223 (48%) Pharmacists: 76 (16.3%) Non-medical professionals: 62 (13.3%) Physiotherapists: 31 (6.7%)
[35]	Turkey	n = 506	297 (58%)	26–35: 169 (33%) 36–44: 168 (33) 45–60: 153 (30%) >60: 16 (4%)	Pediatricians: 506 (100%)
[36]	France	n = 4349	2806 (77.4%)	<25: 202 (5.6%) 25–40: 1675 (46.2%) 41–50: 908 (25.1%) >50: 838 (23.1%) Missing: 29 (16.7%)	Frontline caregivers: 1940 (53.6%) Other caregivers: 1018 (28.1%) Administrative and non-caregiver staff: 624 (17.3%) Unclassified: 35 (1.0%) Missing: 730 (16.8%)
[44]	USA	n = 415	-	-	Medical students: 163 (39%) Dental students: 245 (59%)
[47]	Barbados	n = 343	260 (76%)	18–34: 144 (42%) >35: 199 (58%)	Medical Doctors: 119 (34.7%) Nurses: 144 (42%) Allied health/Admin: 80 (23.3%)
[45]	USA	n = 168	96 (57%)	-	Medical students: 168 (100%)
[46]	USA	n = 248	-	Mean age: 26.3 ± 3.8 (SD)	Dental students: (100%)
[29]	Saudi Arabia	n = 674	324 (48.1%)	18–29: 392 (58.2%) 30–49: 251 (37.2%) 50–64: 27 (4%) >64: 4 (0.6%)	Medical doctors: 76 (11.3%) Dentists: 191 (28.3%) Nurses: 41 (6.1%) Pharmacists: 29 (4.3%) Dental assistants/Hygienists: 6 (0.9%) Dental technicians: 6 (0.9%) Healthcare students: 238 (35.3%) Other health professionals: 87 (12.9%)
[33]	Turkey	n = 1808	1227 (68.1%)	18–35: 780 (43.3%) 36–50: 664 (36.9%) >50: 357 (19.8%)	Physicians: 927 (51.5%), Nurses and midwives: 479 (24.6%) Medical technicians: 80 (4.4%) Aides or helpers: 93 (5.2%) Other: 222 (12.3%)
[42]	Italy	n = 2137	1528 (71.70%)	<31: 190 (8.92%) 31–40: 440 (20.65%) 41–50: 571 (26.79%) 51–60: 700 (32.85%) >60: 230 (10.79%)	Medical Doctors: 634 (29.91%) Nurses: 894 (42.17%) Auxiliary nurses: 100 (4.72%) Technicians: 189 (8.92%) Pharmacists: 24 (1.13%) Territorial medicine: 74 (3.50%) Administration: 111 (5.26%) Other: 64 (3.03%)
[28]	Saudi Arabia	n = 673	268 (39.82%)	18–29: 147 (21.84%) 30–39: 305 (45.32%) 40–49: 141 (20.95%) 50–59: 56 (8.32%) ≥60: 24 (3.57%)	Frontline healthcare worker: • Yes: 327 (48.59%) • No: 346 (51.41%)
[41]	Italy	n = 166	99 (59.6%)	Mean age: 49.1 ± 10.7 (SD) <50: 106 (63.9%) >50: 60 (36.1%)	Occupational Physicians: 166 (100%)
[38]	Austria	n = 612	Female: 444 (73.15%) Male: 160 (26.36%) Diverse: 3 (0.49%)	Mean age: 42.7 ± 10.56 (SD)	Nursing and social care employees: 100%
[40]	Czech Republic	n = 240	73.75%	20–23 years: 58.3% 24–29 years: 41.7%	Dental students: 240 (100%)
[30]	Saudi Arabia	n = 1285	822 (64%)	25–34: 434 (33.8%) 35–44: 477 (37.1%) 45–54: 273 (21.2%) ≥55: 101 (7.9%)	Physician: 162 (68.1%) Other: 76 (31.9%)
[48]	Egypt	n = 455	367 (80.7%)	18–24: 51 (11.2%) 25–35: 182 (40%) 36–45: 132 (29%) 46–60: 86 (18.9%) >60: 4 (0.9%)	Physicians: 118 (25.9%) Nurses: 172 (37.8%) Dentists: 6 (1.3%) Pharmacists: 10 (2.2%) Administrators: 46 (10.1%) Radiology or laboratory technicians: 20 (4.4%) Workers or security officers: 24 (5.3%) Other: 59 (13%)
[39]	Slovenia	n = 832	-	-	HCWs
[37]	United Kingdom	n = 3235	2705 (74.3%)	16–40: 1020 (31.5%) 40–55: 1239 (38.3%) >55: 963 (29.8%)	Medical staff: 778 (24.1%) Nursing staff: 698 (21.6%) Allied health professionals: 917 (28.4%) Pharmacy staff: 62 (1.9%) Healthcare scientists: 146 (4.5%) Ambulance staff: 94 (2.9%) Dental staff: 93 (2.9%) Optical staff: 82 (2.5%) Admin/estates/other staff: 184 (5.7%) Missing: 103 (3.2%)
[34]	Turkey	n = 320	232	Mean age: 37.0 ± 10.0 (SD)	HCWs

**Table 2:** Demographic features of the participants included in the meta-analysis: HCWs’ acceptance of COVID-19 vaccination mandates for HCWs.

Study	Country	Participants	Gender (Female)	Age (Years)	Profession
[61]	USA	n = 209	Female: 170 (81%) Male: 37 (18%) Transgender/non-binary: 2 (1%)	18–35: 85 (41%) 36–45: 58 (28%) 46–55: 44 (21%) ≥56: 23 (10%)	Advanced practice providers (NP, CNM, PA): 14 (7%) Emergency medical technicians/paramedics: 18 (9%) Medical or patient care assistants: 14 (7%) Nurses (RN, LPN): 38 (18%) Physicians: 71 (34%) Social workers/mental health specialists: 14 (7%) Other: 40 (19%)
[50]	Italy	n = 465	225 (48%)	Mean age: 51 ± 9 (SD)	Physicians: 212 (45.6%) Nurses: 120 (25.8%) Healthcare technicians: 41 (8.8%) Administrative/other: 92 (19.8)
[49]	Sudan	n = 930	626 (67.3%)	Mean age: 28.7 ± 6.7 (SD)	Anesthesiologists: 2 (0.2%) Dentists: 92 (9.9%) Doctors: 585 (62.9%) Medical laboratory staff: 70 (7.5%) Nurses: 27 (2.9%) Pharmacists: 118 (12.7%) Physiotherapists: 4 (0.4%) Radiologists: 6 (0.6%) Staff in medical universities: 26 (2.8%)
[53]	Greece	n = 1132	804	Mean age: 43.1	Physicians: 428 (38%) Nurses: 515 (45%) Other: 189 (17%)
[43]	Italy	n = 4677	Female: 3132 (67.0%) Male: 1475 (31.5%) Not specified: 65 (1.4%) Other: 5 (0.1%)	18–30: 389 (8.3%) 31–40: 866 (18.5%) 41–50: 1169 (25%) 51–60: 1380 (29.5%) >60: 873 (18.7%)	Health professionals: 2474 (52.9%) Nursing health professionals: 1230 (26.3%) Obstetric health professionals: 79 (1.7%) Rehabilitation health professionals: 482 (10.3%) Technical health professionals: 179 (3.8%) Prevention health professionals: 29 (0.6%) Health operators: 204 (4.4%)
[32]	Cyprus	n = 504	320 (63%)	Mean age: 36.7 ± 9.6 (SD)	Physicians: 62 (13.3%) Nursing staff: 223 (48%) Pharmacists: 76 (16.3%) Non-medical professionals: 62 (13.3%) Physiotherapists: 31 (6.7%)
[58]	USA	n = 1047	515 (49%)	-	Physicians: 747 (71%) Other: 300 (29%)
[66]	India	n = 1068	519 (48%)	-	Medical students: 1068 (100%)
[36]	France	n = 4349	2806 (77.4%)	<25: 202 (5.6%) 25–40: 1675 (46.2%) 41–50: 908 (25.1%) >50: 838 (23.1%) Missing: 29 (16.7%)	Frontline caregivers: 1940 (53.6%) Other caregivers: 1018 (28.1%) Administrative and non-caregiver staff: 624 (17.3%) Unclassified: 35 (1.0%) Missing: 730 (16.8%)
[57]	Poland	n = 1051	830 (77%)	Mean age: 26.8 ± 9.7 (SD) 19–26: 815 (75.5%) >27: 260 (24.1%) Missing: 5 (0.5%)	Medical doctors: 135 (12.5%) Nurses and midwives: 128 (11.8%) Medical students: 423 (39.2%) Students of nursing and midwifery: 394 (36.5%)
[67]	Pakistan	n = 208	118 (57%)	Mean age: 27.78 ± 11.19	HCWs
[62]	Australia	n = 3074	2532 (82%)	18–49: 1643 (55.4%) >50: 1321 (44.6%)	Medical Doctors: 171 (5.6%) Nurses: 2071 (67.4%) Pharmacists: 53 (1.7%) Allied health professionals: 232 (7.5%) Personal support staff: 66 (2.1%) Ambulance staff: 124 (4.0%) Other: 357 (11.6%)
[63]	Australia	n = 252	178 (70%)	18–29: 26 (11.7%) 30–49: 73 (32.9%) 50–64: 109 (49.1%) >65: 14 (6.3%)	Disability support workers: 252 (100%)
[44]	USA	n = 415	-	-	Medical students: 163 (39%) Dental students: 245 (59%)
[69]	Tanzania	n = 811	388 (48%)	Mean age: 35 ± 9.04	Cadre Medical attendants: 105 (12.9%) Nurses/clinical officers: 419 (51.7%) Doctors/specialists: 287 (35.4%)
[45]	USA	n = 168	96 (57%)	-	Medical students: 168 (100%)
[54]	Greece	n = 134	92 (68%)	-	Dental students: 134 (100%)
[55]	Greece	n = 1284	816 (63%)	≤30: 214 (16.7%) 31–40: 317 (24.7%) 41–50: 384 (29.9%) >50: 367 (28.6%)	Physicians: 402 (31.3%) Nursing personnel: 470 (36.6%) Paramedical personnel: 142 (11.1%) Administrative personnel: 170 (13.2%) Supportive personnel: 94 (7.3%) Unknown: 6 (0.5%)
[46]	USA	n = 248	-	Mean age: 26.3 ± 3.8 (SD)	Dental students: (100%)
[59]	USA	n = 1899	1221 (64.3%)	<25: 649 (34.18%) 25–29: 1091 (57.45%) >30: 159 (8.37%)	Medical students: 1899 (100%)
[56]	France	n = 1964	1532 (78%)	18–29: 306 (16%) 30–49: 1.118 (57%) >50: 540 (27%)	Physicians: 423 (21.5%) Paramedical staff: 876 (44.6%) Administrative workers: 432 (22.0%) Technical staff: 213 (10.8%) Other: 20 (1.0%)
[52]	Italy	n = 130	97 (74.6%)	≤30: 24 (18.5%) 31–40: 29 (22.3%) 41–50: 34 (26.2%) 51–60: 39 (30.0%) >60: 4 (3.1%)	Physicians: 38 (29%) Nurses: 58 (44%) Other HCWs: 34 (27%)
[51]	Italy	n = 2142	Female: 1125 (52.5%) Male: 1007 (47.0%) Not specified: 10 (0.5%)	15–25: 40 (1.9%) 26–35: 327 (15.3%) 36–45: 299 (14.0%) 46–55: 399 (18.6%) 56–65: 601 (28.0%) 66–75: 436 (20.3%) 76–85: 36 (1.7%) 86–95: 4 (0.2%)	Physicians: 1538 (71.8%) Dentists: 171 (8.0%) Nurses: 275 (12.8%) Chemists: 3 (0.1%) Healthcare assistants: 19 (0.9%) Other: 136 (6.4%)
[40]	Czech Republic	n = 240	73.75%	20–23: 58.3% 24–29: 41.7%	Dental students: 240 (100%)
[60]	USA	n = 3479	Female: 2598 (75%) Male: 864 (25%) Trans/Gender non-binary/not specified above: 7 (0.2%) Do not wish to reply: 10 (0.3%)	18–30: 816 (23%) 31–40: 1061 (30%) 41–50: 686 (20%) 51–60: 571 (16%) 61–70: 326 (9.4%) >70: 19 (0.5%)	Direct Patient Care Providers (DPCPs): 1573 (45%) Direct medical providers (DMPs): 1207 (35%) Administrative staff working in hospitals without direct patient contact: 295 (8.5%) Others without direct patient contact: 404 (12%)
[65]	India	n = 254	72 (28.3%)	-	Medical doctors: 172 (67.7%) Paramedical workers: 82 (32.3%)
[48]	Egypt	n = 455	367 (80.7%)	18–24: 51 (11.2%) 25–35: 182 (40%) 36–45: 132 (29%) 46–60: 86 (18.9%) more than 60: 4 (0.9%)	Physicians: 118 (25.9%) Nurses: 172 (37.8%) Dentists: 6 (1.3%) Pharmacists: 10 (2.2%) Administrators: 46 (10.1%) Radiology or laboratory technicians: 20 (4.4%) Workers or security officers: 24 (5.3%) Other: 59 (13%)
[64]	Mongolia	n = 238	195 (81.9%)	18–25: 18 (7.6%) 26–35: 148 (62.2%) 36–45: 48 (20.2%) 46–55: 20 (8.4%) >55: 4 (1.7%)	Physicians: 162 (68.1%) Other: 76 (31.9%)
[68]	Hong Kong, Nepal, Vietnam	n = 3396	2589 (76.2%)	18–29: 560 (16.5%) 30–39: 1058 (31.2%) 40–49: 834 (24.6%) ≥50: 928 (27.3%)	Nurses: 2636 (77.6%) Doctors: 760 (22.4%)
[37]	United Kingdom	n = 3235	2705 (74.3%)	16–40: 1020 (31.5%) 40–55: 1239 (38.3%) >55: 963 (29.8%)	Medical staff: 778 (24.1%) Nursing staff: 698 (21.6%) Allied health professionals: 917 (28.4%) Pharmacy staff: 62 (1.9%) Healthcare scientists: 146 (4.5%) Ambulance staff: 94 (2.9%) Dental staff: 93 (2.9%) Optical staff: 82 (2.5%) Admin/estates/other staff: 184 (5.7%) Missing: 103 (3.2%)

**Table 3:** Comparison between the results of the primary analysis and the sensitivity analyses.

	HCWs for the General Population	HCWs for HCWs
Primary analysis	50% (95% CI: 38%, 61%)	64% (95% CI: 55%, 72%)
Alternative dichotomization—sensitivity analysis	56% (95% CI: 43%, 67%)	68% (95% CI: 59%, 75%)
Risk of bias assessment—sensitivity analysis	45% (95% CI: 30%, 60%)	55% (95% CI: 41%, 69%)

**Table 4:** Sub-group analyses’ results—HCWs’ acceptance towards COVID-19 Mandatory vaccination.

Sub-Groups	Percentage (95% CI)
*By W.H.O. regions for HCWs*	
African and Eastern Mediterranean Region	57% (33%, 78%)
Western Pacific and South-East Asia Region	67% (57%, 75%)
European Region	59% (40%, 76%)
Region of the Americas	70% (54%, 82%)
*By W.H.O. regions for the general population*	
Eastern Mediterranean Region	63% (47%, 76%)
Region of the Americas	46% (32%, 60%)
European Region	44% (28%, 62%)
*By year of publication for HCWs*	
2021	56% (47%, 65%)
2022	69% (54%, 80%)
*By year of publication for the general population*	
2021	48% (34%, 63%)
2022	51% (34%, 68%)

## Data Availability

Detailed methods, results, and additional data are available in the manuscript and the correspondent Supplementary Materials.

## Supplementary Materials

The following supporting information can be downloaded at: critical appraisal of individual studies https://www.mdpi.com/article/10.3390/vaccines11040880/s1. References [18,28,29,30,31,32,33,34,35,36,37,38,39,40,41,42,43,44,45,46,47,48,49,50,51,52,53,54,55,56,57,58,59,60,61,62,63,64,65,66,67,68,69,70,71,72,73,74,75,76,77,78,79,80,81,82,83,84] are cited in Supplementary Materials.
