# Supplementary material for: Healthcare Workers’ Attitudes towards Mandatory COVID-19 Vaccination: A Systematic Review and Meta-Analysis
Source: Vaccines (Basel). 2023 Apr 21;11(4):880. doi: 10.3390/vaccines11040880 (PMC10142794; doi:10.3390/vaccines11040880)
Supplement: Supplementary file 1 [file vaccines-11-00880-s001.zip › vaccines-2285928-supplementary.pdf]

Critical appraisal of individual studies [1]								
**Study and setting**	**Sample size (n)**	**Sample design**	**Sampling frame**	**Measures**	**Unbiased assessors**	**Response rate and refusers**	**Prevalence rates**	**Score and limitations**
Aldosary (2021) [2],<br><br>Saudi Arabia	334	Target population: All nurses working in the government hospital and governmental primary health care centres in Ar-Rass	All nurses registered in general authority for statistics<br><br>The data collection period was from 27/11/2020 to 7/12/2020	online self-administered questionnaire<br><br>Cronbach's alpha: 0.754	No	71.4%<br><br>Refusers not described	Active nurses<br><br>78.7% of participants agree with COVID-19 mandatory vaccinations once available<br><br>No Confidence Intervals provided	**Score: 5**<br><br>Biased sampling frame (there is no description of how the HCWs were contacted)<br><br>Refusers not described<br><br>No unbiased assessor<br><br>No Confidence Intervals provided
Aloweidi (2021) [3],<br><br>Jordan	646	Target population: "Medical field workers from Jordan University Hospital with direct contact with patients at inpatient or outpatient departments"	Sampling frame not described<br><br>The survey was conducted between 22 January 2021 and 28 February 2021	online structured self-administered questionnaire<br><br>Cronbach's alpha value was 0.76	No	82%<br><br>Refusers not described	Medical field HCWs<br><br>25.4% acceptance for COVID-19 vaccine mandates in schools, universities, and workplaces<br><br>33.1% acceptance for COVID-19 vaccine mandates for travelling<br><br>No Confidence Intervals provided	**Score: 5**<br><br>Refusers not described<br><br>No unbiased assessor<br><br>No Confidence Intervals provided<br><br>Only HCWs in direct contact with patients included
Arif (2022) [4],<br><br>Saudi Arabia	529	Target population: All HCWs at King Saud University Medical City<br><br>Purposive sample	"Online questionnaire distributed through social networking sites such as WhatsApp, Google, and Twitter between May 1 and September 30, 2021"	Online questionnaire<br><br>No assessment of the internal consistency of the measurement	No	Response rate not described<br><br>Refusers not described	Active HCWs<br><br>27% decreased odds of vaccine acceptance when COVID-19 vaccine was a mandate<br><br>No Confidence Intervals provided<br><br>No Confidence Intervals provided	**Score: 3**<br><br>Biased sampling frame (social network list)<br><br>Response rate not described<br><br>No assessment of the internal consistency of the measurement

								Refusers not described<br><br>No unbiased assessor<br><br>No Confidence Intervals provided
Badahdah (2022) [5],<br><br>Oman	346	Target population not described	Samling frame not described	Web-based survey<br><br>Cronbach's alpha coefficient was 0.87	No	Response rate not described<br><br>Refusers not described	Unvaccinated physicians and nurses working in Oman<br><br>Male and young HCWs more prone to accept COVID-19 vaccine mandates $F (1, 341) = 5.77$; $p = 0.020$, $F (1, 341): 5.71$; $p = 0.020$<br><br>No Confidence Intervals provided	**Score: 3**<br><br>Target population not described<br><br>Samling frame not described<br><br>No unbiased assessor<br><br>Refusers not described<br><br>Response rate not described<br><br>No Confidence Intervals provided
Casey (2022) [6],<br><br>USA	209	"English-speaking, at least 18 years old, and worked in healthcare during the COVID-19 pandemic"	"The survey was distributed using the snowball sampling method, where Advisory Board members and the study team shared the survey link with potential participants from their networks"	Web-based survey<br><br>"The survey was pilot tested by 8 healthcare workers and revised before dissemination"	No	Response rate not described<br><br>Refusers not described	"English-speaking, at least 18 years old, and worked in healthcare during the COVID-19 pandemic"<br><br>COVID-19 vaccine mandates in healthcare settings: 91.9% agree (full or under conditions support)<br><br>No Confidence Intervals provided	**Score: 3**<br><br>Sample size < 300<br><br>Biased sampling frame (personal contacts)<br><br>No unbiased assessor<br><br>Refusers not described<br><br>Response rate not described<br><br>No Confidence Intervals provided
Ciliberti (2022) [7],<br><br>Italy	240	Target population: All students who were enrolled in the third year of the School of Medicine, on a core lecture	"The questionnaire was administered via the e-learning platform of Genoa University (AulaWeb) during December 2021"	online, anonymous questionnaire<br><br>Cronbach's Alpha of 0.777	No	240 out of 284 (84%)<br><br>Refusers not described	Medical students<br><br>students believed that: "COVID-19 vaccine mandates should be made obligatory for the whole community" and for "all students",	**Score: 5**<br><br>sample size < 300<br><br>No unbiased assessor<br><br>Refusers not described<br><br>No Confidence Intervals

							and even that “those who refuse vaccination against COVID-19 should be excluded from university”, 8-10 (Likert-type answers medians)”<br><br>No Confidence Intervals provided	provided
Constantino (2022) [8],<br><br>Italy	1,450	Target population: All Pharmacists of the Palermo Province Pharmacists’ Order	“The administration of the two questionnaires took place through dedicated links created on the Google Documents® platform, with access reserved for CPs on the restricted area of the website of the Palermo Province Pharmacists’ Order”	Online questionnaire<br><br>Cronbach’s alpha was 0.87	No	83.5%<br><br>Refusers not described	Community Pharmacists<br><br>64.3% of those who changed their opinion regarding Covid-19 vaccination, they did it due to vaccines mandates<br><br>No Confidence Intervals provided	**Score: 6**<br><br>No unbiased assessor<br><br>Refusers not described<br><br>No Confidence Intervals provided
Craxi (2021) [9],<br><br>Italy	465	All HCWs of the University Hospital P. Giaccone of Palermo	Access to a web-based simulation platform provided by the Hospital information system	Web-based questionnaire<br><br>No assessment of the internal consistency of the measurement	No	465 out of 2068 (22.5%)<br><br>Refusers not described	Active HCWs<br><br>Preferred vaccination strategy for healthcare workers: mandatory answered vaccination 229 out of 465<br><br>No Confidence Intervals provided	**Score: 4**<br><br>Inadequate response rate (< 70%)<br><br>Refusers not described<br><br>No unbiased assessor<br><br>No Confidence Intervals provided<br><br>No assessment of the internal consistency of the measurement
Elbadawi (2022) [10],<br><br>Sudan	930	Target population: All healthcare workers who were working at hospitals in Sudan	“The questionnaire was distributed as a Google Form document through multiple social media platforms (Facebook, Twitter, WhatsApp, Telegram), non-governmental organizations (NGOs) and academic	Web-based questionnaire<br><br>Cronbach’s alpha: 0.790	No	Response rate not described<br><br>Refusers not described	Active HCWs<br><br>Acceptance of mandatory vaccinations for the general population: 470/852 and for HCWs: 636/852	**Score: 4**<br><br>Biased sampling frame (social network contacts)<br><br>No unbiased assessor<br><br>Response rate not

			institutions"				No Confidence Intervals provided	described<br><br>Refusers not described<br><br>No Confidence Intervals provided
Fotiadis (2021) [11],<br><br>Greece	1,456	Target population: All HCWs in Greek public hospitals<br><br>Random-geographically stratified sampling	Questionnaires were disseminated proportionally in each hospital (20 hospitals in total) in May 2021	Anonymized paper-based<br><br>Cronbach's alpha value of 0.70	No	41%<br><br>Refusers not described	Active HCWs<br><br>COVID-19 mandatory vaccination acceptance for HCWs: proportional ratio 1.56 (1.47–1.65)	**Score: 5**<br><br>Sampling frame not described in detail (how and when the questionnaire was disseminated?)<br><br>No unbiased assessor<br><br>Inadequate response rate (< 70%)
Ghirotto (2022) [12],<br><br>Italy	4,677	Target population: HCWs who were practicing a health profession recognized by the Italian State as subject to mandatory vaccination. Actively working in public or private healthcare structures	"Authors disseminated the link to the questionnaire among their professional contacts (also via social networks), The Italian Order of Doctors, Surgeons, and Orthodontists disseminated the questionnaire to their associates"	Online questionnaire<br><br>No objective assessment of the measurement	No	Response rate not described<br><br>Refusers not described	Active HCWs<br><br>mandatory vaccination helpful for making your workplace safer for everyone: 3,705/4,071<br><br>mandatory vaccination helpful for protecting public health: 3,773/4,071<br><br>No Confidence Intervals provided	**Score: 3**<br><br>Biased sampling frame (social network and personal contacts)<br><br>No objective assessment of the measurement<br><br>No unbiased assessor<br><br>Response rate not described<br><br>Refusers not described<br><br>No Confidence Intervals provided
Giannakou (2022) [13],<br><br>Cyprus	504	Target population: "Greek-Cypriot HCWs working in either public or private service provision, aged 18 years old and above, and living in the five government-controlled municipalities of the	"The online questionnaire was administered using Google Forms and dispersed using instant messaging apps, social media platforms, and social networking sites"	Online questionnaire<br><br>Cronbach's alpha coefficient ranged between 0.63 and 0.96	No	Response rate not described<br><br>Refusers not described	Active HCWs<br><br>COVID-19 vaccination should be mandatory for healthcare professionals: agree 247/504<br><br>No Confidence Intervals provided	**Score: 4**<br><br>Non-probability sampling<br><br>Biased sampling frame (social network contacts)<br><br>No unbiased assessor<br><br>Response rate not described

		Republic of Cyprus”<br><br>Non probability convenience sampling						Refusers not described<br><br>No Confidence Intervals provided
Gönüllü (2021) [14],<br><br>Turkey	506	Target population: All HCWs in The Turkish Pediatric Workshop group in Telegram app	Google Forms link was sent to the members of Turkish Paediatric Workshop group	Online questionnaire<br><br>No objective appraisal of the measurement tool	No	22%<br><br>Refusers not described	Active HCWs<br><br>COVID-19 vaccine should be mandatory: 303/506<br><br>No Confidence Intervals provided	**Score: 3**<br><br>Biased sampling frame (selection bias – HCWs not in the telegram group were excluded)<br><br>No objective appraisal of the measurement tool<br><br>No unbiased assessor<br><br>Inadequate response rate (< 70%)<br><br>Refusers not described<br><br>No Confidence Intervals provided
Grabert (2022) [15],<br><br>USA	1,047	Target population: US PCPs who were members of a standing national panel maintained by a survey research company	The survey company emailed invitations to panel members	Online questionnaire<br><br>No objective appraisal of the measurement tool	No	61%<br><br>Refusers not described	Primary Care HCWs<br><br>No. of PCPs who supported Covid-19 vaccine mandates: 871/1,047<br><br>No Confidence Intervals provided	**Score: 4**<br><br>Biased sampling frame (only HCWs of the national panel were included)<br><br>No objective appraisal of the measurement tool<br><br>Inadequate response rate (< 70%)<br><br>No unbiased assessor<br><br>Refusers not described<br><br>No Confidence Intervals provided
Jain (2021) [16],<br><br>India	1,068	Target population: All medical students in India<br><br>Respondent-driven	“The link was shared by the student investigator within the social media network of medical student	Online questionnaire<br><br>No objective appraisal of the	No	Response rate not described<br><br>Refusers not described	Medical students<br><br>COVID-19 vaccine should be made mandatory for the	**Score: 3**<br><br>Biased sampling frame (students without this particular social media

		sampling strategy	The students further circulated it among their acquaintances within the same medical college”	measurement tool			health care workers: 800/1068<br><br>No Confidence Intervals provided	network?)<br><br>Non random sampling<br><br>No objective appraisal of the measurement tool<br><br>No unbiased assessor<br><br>Response rate not described<br><br>Refusers not described<br><br>No Confidence Intervals provided
Jankowska-Polanska (2022) [17], Poland	497	Target population not described	Not described	Online questionnaire<br><br>Pre-validated<br><br>Cronbach's alpha coefficient 0.75	No	71%<br><br>Refusers not described	workers in a medical centre or medical students<br><br>Do you think healthcare professionals should be compulsorily vaccinated against COVID-19: agree median 4 Q3–5<br><br>No Confidence Intervals provided	**Score: 3**<br><br>Sampling population not descibed<br><br>Samping frame not described<br><br>No unbiased assessor<br><br>Response rate not described<br><br>Refusers not described<br><br>No Confidence Intervals provided
Janssen (2021) [18],<br><br>France	4,349	Target population: Healthcare workers of 21 French institutions (public and private hospitals, and nursing homes)	“The institutions sent e-mail notification to their stuff, advertised posters within the institution, or added an advertising notice to the monthly pay slip”	Online self-questionnaire<br><br>It was pilot tested for clarity, length, and validity with 20 healthcare workers.	No	4,349/44,975 (9.7%)<br><br>Refusers not described	Active HCWs<br><br>COVID-19 vaccination should be mandatory for healthcare workers: 1,138/4,349<br><br>COVID-19 vaccination should be mandatory for general population: 787/4,349<br><br>No Confidence Intervals provided	**Score: 5**<br><br>No unbiased ass6essor<br><br>Inadequate response rate (<70%)<br><br>Refusers not described<br><br>No Confidence Intervals provided
Kalucka (2022)	1,080	Target population:	1,200 questionnaires were	paper-based	No	90%	Vaccinated healthcare	**Score: 5**

[19],<br><br>Poland		Healthcare professionals (doctors, nurses, midwives) working in the academic centres (hospitals and clinics), and students majoring in medicine, nursing, and midwifery studying in Lodz	distributed from 15 May to 15 July 2021	questionnaire<br><br>One month before the main study, a pilot study that analyzed 100 randomly selected healthcare workers was conducted<br><br>The questionnaires were validated on the basis of standard procedures		Refusers not described	workers<br><br>If the COVID-19 vaccination was recommended to be taken every year, should it be obligatory for the health professionals in your opinion? Agree: 760/1051<br><br>No Confidence Intervals provided	Sampling frame not described (how and when were the questionnaires distributed?)<br><br>No unbiased ass6essor<br><br>Refusers not described<br><br>No description of the measurement assessment<br><br>No Confidence Intervals provided
Kashif (2021) [20],<br><br>Pakistan	208	Target population: HCWs aged 18 years and above, residing in Pakistan<br><br>Convenience sampling	A self-administered questionnaire distributed via social media platforms and direct e-mail solicitation	online questionnaire<br><br>The draft questionnaire was pilot tested among 30 participants	No	Response rate not described<br><br>Refusers not described	Active HCWs<br><br>Willingness to get COVID-19 vaccine if it's mandatory in profession: 185/208<br><br>No Confidence Intervals provided	**Score: 2**<br><br>Inadequate sample size<br><br>Biased sampling frame (excluded nonusers of social media platforms)<br><br>Not objective assessment of the measurement<br><br>No unbiased ass6essor<br><br>Response rate not described<br><br>Refusers not described<br><br>No Confidence Intervals provided
Kaufman (2021) [21],<br><br>Australia	3,074	Target population: HCWs living in Victoria<br><br>Snowballing recruitment	"Research advertisements across health services, clinical colleges, councils, associations, unions, networks, and Facebook were used to recruit participants between 12 February and 26 March 2021"	Online questionnaire	No	Response rate not described<br><br>Refusers not described	Active HCWs living in Victoria<br><br>COVID-19 vaccination should be mandated for all healthcare workers: agree 1,544/3,061	**Score: 4**<br><br>No unbiased ass6essor<br><br>Response rate not described<br><br>Refusers not described<br><br>No Confidence Intervals

							No Confidence Intervals provided	provided
Kavanagh (2022) [22],<br><br>Australia	252	Target population: Disability support workers across Australia, 18 years of age or older	Link to the survey sent by disability services, unions and disseminated via social media.<br><br>"The survey was in English and administered online via the RedCap platform and participants were entered into a prize draw"	Online questionnaire<br><br>Measurement tool not objectively assessed (validity, reliability)	No	Response rate not described<br><br>Refusers not described	Active HCWs<br><br>Mandating for DSWs: 109/252, 43.3% (95% CI: (37.2, 49.5)	**Score: 3**<br><br>Biased sampling frame (did all the DWS receive the link? prize draw)<br><br>Inadequate sample size (<300)<br><br>No unbiased ass6essor<br><br>Measurement tool not objectively assessed (validity, reliability)<br><br>Response rate not described<br><br>Refusers not described
Kelekar (2021) [23],<br><br>USA	415	Target population: students from 3 US dental schools and 1 US medical school	All students at these schools were e-mailed a link to the Qualtrics XM online survey	Online questionnaire<br><br>Measurement tool not objectively assessed (validity, reliability)	No	Inadequate response rate (<70%)<br><br>Refusers not described	Active HCWs<br><br>The COVID-19 vaccination should be mandatory for the general public: 67.9% of MS and 40.3% of DS<br><br>The COVID-19 vaccination should be mandatory for all HCPs 85.9% of MS and 53.9% of DS<br><br>No Confidence Intervals provided	**Score: 4**<br><br>No unbiased ass6essor<br><br>Measurement tool not objectively assessed (validity, reliability)<br><br>Inadequate response rate (< 70%)<br><br>Refusers not described<br><br>No Confidence Intervals provided
Konje (2022) [24],<br><br>Tanzania	811	Target population: Health professionals from 23 health facilities in five districts of Tanzania<br><br>Convenience sample	Convenience sample of health professionals who were available at the health facility during data collection period responded to the structured questions<br><br>The data collection procedure was conducted within the	Paper based questionnaire<br><br>structured questionnaire that was adopted from the WHO vaccine	No	811/2,500<br><br>Refusers not described	Active HCWs<br><br>COVID-19 vaccine to be mandatory to all health professionals: 33.54% of HCWs agree<br><br>No Confidence Intervals provided	**Score: 4**<br><br>Nonrandom sampling<br><br>Inadequate response rate (<70%)<br><br>No unbiased ass6essor<br><br>Refusers not described

			health facility premises at different units/departments	acceptance scale				No Confidence Intervals provided
Krishnamurthy (2021) [25],<br><br>Barbados	343	Target population: HCWs of the Queen Elizabeth Hospital (QEH), Barbados<br><br>Purposive sampling of all HCPs working at the QEH	Email invitations were sent to a Google Forms link to the survey during the period of February 14 to 27, 2021	online questionnaire<br><br>Measurement tool not objectively assessed (validity, reliability)	No	343/850<br><br>Refusers not described	Active HCWs<br><br>COVID-19 vaccination should be compulsory, once available: 101, 29.5%<br><br>No Confidence Intervals provided	**Score: 3**<br><br>Biased sampling frame (which e-mail list was used?)<br><br>Measurement tool not objectively assessed (validity, reliability)<br><br>No unbiased ass6essor<br><br>Inadequate response rate (<70%)<br><br>Refusers not described<br><br>No Confidence Intervals provided
Lee (2022) [26],<br><br>USA	12,875	"Sample of The National Immunization Survey Adult COVID Module a nationally representative survey with approximately 60,000 adult respondents (aged 18 years and older)"<br><br>HCWs from 50 states and the District of Columbia excluding Puerto Rico and the U.S. Virgin Islands	"Telephone interviews from a random digit dialled sample of cell telephone numbers stratified by state and the District of Columbia as well as Puerto Rico and the U.S. Virgin Islands"	telephone interviews<br><br>Measurement tool not objectively assessed (validity, reliability)	Interviewe r	20.5% - 20.9%<br><br>Refusers not described	90.5% (95%CI 88.7 – 92.1) of HCWs who faced working requirements had been vaccinated against COVID-19, as compared to 73.3% (95%CI 71.1 – 75.3) of HCP without vaccination requirements (24% increased odds, 95%CI 1.19 – 1.28)	**Score: 5**<br><br>Measurement tool not objectively assessed (validity, reliability)<br><br>Inadequate response rate (<50%)<br><br>Refusers not described
Lucia (2021) [27],<br><br>USA	168	Target population: Medical students at a single allopathic medical school in Southeast Michigan	Not described	online survey<br><br>Measurement tool not objectively assessed	No	34%<br><br>Refusers not described	Active HCWs<br><br>The COVID-19 vaccination should be mandatory for the public: agree 110	**Score: 2**<br><br>Inadequate sample size<br><br>Biased sampling (No description)

				(validity, reliability)			(67.9%)<br><br>The COVID-19 vaccination should be mandatory for all health care providers 140 (85.9%)	Not objective assessment of the measurement<br><br>No unbiased ass6essor<br><br>Inadequate response rate<br><br>Refusers not described<br><br>No Confidence Intervals provided
Maltezou (2021) [28],<br><br>Greece	1,571	Target population: HCWs in eight tertiary-care hospitals across Greece	"A printed questionnaire (in Greek language) was distributed to HCP, regardless of their characteristics, during their work shift by the Infection Control nurses Committee of each hospital from September 1 through October 31, 2020"	Printed questionnaire<br><br>Measurement tool not objectively assessed (validity, reliability)	No	14.5%<br><br>Refusers not described	Active HCWs<br><br>HCWs who supported COVID-19 vaccine mandates for HCWs were more prone to get vaccinated (83.9%) against COVID-19 in relation to those who not supported COVID-19 vaccine mandates (19%) for HCWs<br><br>No Confidence Intervals provided	**Score: 4**<br><br>Measurement tool not objectively assessed (validity, reliability)<br><br>No unbiased ass6essor<br><br>Inadequate response rate<br><br>Refusers not described<br><br>No Confidence Intervals provided
Maltezou (2022) [29],<br><br>Greece	134	Target population: Undergraduate students at the Dental School of the University of Athens	"A web-based link was distributed to all undergraduate dental students via an inviting email sent from the Administration Office of the Dental School"	Online questionnaire<br><br>Measurement tool not objectively assessed (validity, reliability)	No	134/600<br><br>Refusers not described	Active HCWs<br><br>57.1% of the 123 students who favoured mandatory vaccinations for dentists, accepted COVID-19 vaccine mandates<br><br>No Confidence Intervals provided	**Score: 3**<br><br>Inadequate sample size<br><br>Measurement tool not objectively assessed (validity, reliability)<br><br>No unbiased ass6essor<br><br>Inadequate response rate<br><br>Refusers not described<br><br>No Confidence Intervals provided
Maltezou (2022) [30],<br><br>Greece	1,284	Target population: HCWs in Eight tertiary-care hospitals across Greece	"The questionnaire was distributed to a total of 1,879 out of 9,356 HCP by the Infection Control Committees of participating hospitals"	Paper-based questionnaire<br><br>Measurement tool not	No	1,284/1,879 (68%)<br><br>Refusers not described	Active HCWs<br><br>Of the 1,051 HCPs who favoured mandatory vaccinations for HCP,	**Score: 3**<br><br>Non random sampling<br><br>Measurement tool not

				objectively assessed (validity, reliability)			576 (54.8%) believed that all vaccinations should be mandatory for them, while 445 (42.3%) favoured mandatory vaccinations against specific VPDs. 63.5% of the 445 agreed with COVID-19 vaccine mandates<br><br>No Confidence Intervals provided	objectively assessed (validity, reliability)<br><br>No unbiased ass6essor<br><br>Inadequate response rate (< 70%)<br><br>Refusers not described<br><br>No Confidence Intervals provided
Mascarenhas (2021) [31],<br><br>USA	248	Target population: All dental students at three geographically distributed dental schools in Florida, Michigan, and Utah	The survey was administered anonymously online using qualtricsXM	Online questionnaire<br><br>Measurement tool not objectively assessed (validity, reliability)	No	18%<br><br>Refusers not described	The COVID-19 vaccination should be mandatory for the public: 40%<br><br>The COVID-19 vaccination should be mandatory for all health care providers: 53.5%<br><br>No Confidence Intervals provided	**Score: 2**<br><br>Inadequate sample size<br><br>Biased sampling (how were the participants informed? duplicates answers?)<br><br>Not objective assessment of the measurement tool<br><br>No unbiased ass6essor<br><br>Inadequate response rate<br><br>Refusers not described<br><br>No Confidence Intervals provided
Masood (2022) [32],<br><br>Pakistan	331	Target population: HCWs Departments of the Sindh Institute of Urology and Transplantation<br><br>Proportionate probability sampling technique	Not described	"The questionnaire, in both Urdu and English languages, assessing determinants of hesitancy, was designed using the SAGE Vaccine Hesitancy	No	Response rate not described<br><br>Refusers not described	Active HCWs<br><br>59% of the participants answered that official requirements are the reason of getting vaccinated against COVID-19<br><br>No Confidence Intervals provided	**Score: 4**<br><br>Biased sampling (how were the participants informed?)<br><br>No unbiased ass6essor<br><br>No response rate<br><br>Refusers not described<br><br>No Confidence Intervals

				Matrix"<br><br>"The questionnaire was pilot-tested on 30 individuals for validity, and was modified accordingly before administering them individually and in groups"				provided
Mayan (2021) [33],<br><br>USA	1,899	Target population: Students enrolled in a MD or DO program at a U.S. medical school	"Email was sent to the student affairs offices of all 212 U.S. medical schools with request to distribute the website link for the online survey to their students. Additional participation was recruited via medical students targeted social media"	Online questionnaire<br><br>Measurement tool not objectively assessed (validity, reliability)	No	Response rate not described<br><br>Refusers not described	Active HCWs<br><br>Make healthcare worker receiving COVID-19 vaccine mandatory 1096/1,899 (57.71%)<br><br>No Confidence Intervals provided	**Score: 3**<br><br>Biased sampling frame (how many participants received finally the invitation? social media contacts)<br><br>Measurement tool not objectively assessed (validity, reliability)<br><br>No unbiased ass6essor<br><br>No response rate provided<br><br>Refusers not described<br><br>No Confidence Intervals provided
Mustapha (2021) [34],<br><br>Nigeria	440	Target population: Health sciences students from four selected tertiary institutions in North-western Nigeria aged 18 years and above	"The participants were invited by sharing the hyperlink to the survey through emails, social media pages, and groups"<br><br>A convenience sampling with a simplified snowball sampling technique was used to recruit the participants for this study	online self-administered survey<br><br>Cronbach's alpha, was found to be 0.88<br><br>"The study survey tool was developed	No	Response rate not described<br><br>Refusers not described	Active HCWs<br><br>52.3 would get the Covid-19 vaccine if mandated by the heads of institution<br><br>No Confidence Intervals provided	**Score: 4**<br><br>Biased sampling frame (social media contacts list)<br><br>No unbiased ass6essor<br><br>No response rate<br><br>Refusers not described<br><br>No Confidence Intervals

				based on a validated vaccine hesitancy scale provided by the World Health Organization (WHO) Strategic Advisory Group of Experts on Immunization (SAGE) relevant literature search and discussion with experts”				provided
Navarre (2021) [35],<br><br>France	1,964	Target population: French HCWs in 11 AURA (Auvergne-Rhône-Alpes) hospitals	“The Human Resources Department of each facility sent the questionnaire to the professional e-mail addresses of all HCWs on December 7, 2020. A reminder was sent one week later”	Online questionnaire<br><br>Measurement tool not objectively assessed (validity, reliability)	No	Response rate not described<br><br>Refusers not described	Active HCWs<br><br>35.3% of respondents were in favour of mandatory vaccination<br><br>No Confidence Intervals provided	**Score: 4**<br><br>Measurement tool not objectively assessed (validity, reliability)<br><br>No unbiased ass6essor<br><br>No response rates<br><br>Refusers not described<br><br>No Confidence Intervals provided
Noushad (2021) [36],<br><br>SaudiArabia	674	Target population: HCWs in Saudi Arabia	“The survey questionnaire developed on Google Forms was distributed through WhatsApp in the months of February and March 2021”<br><br>“Due to the greater number of HCWs in the central region, majority of the participants were from this region (Not geographically stratified, not representative sample)”	Online questionnaire<br><br>Measurement tool not objectively assessed (validity, reliability)	No	59.9%<br><br>Refusers not described	Active HCWs<br><br>I support a mandatory vaccination program for COVID-19: agree 58.7%<br><br>No Confidence Intervals provided	**Score: 3**<br><br>Biased sampling frame (social media contact list)<br><br>Measurement tool not objectively assessed (validity, reliability)<br><br>No unbiased ass6essor<br><br>Refusers not described<br><br>No Confidence Intervals

								provided
Öncel (2022) [37],<br><br>Turkey	1,808	Target population: Turkish health workers	"Contacted and invited, through the institutional electronic messaging system in a state university hospital by means of e-mail in two private hospitals and in the authors' social network groups"	Online questionnaire<br><br>Measurement tool not objectively assessed (validity, reliability)	No	Response rate not described<br><br>Refusers not described	Active HCWs<br><br>1,052 (58.5%) of the HCWs thought vaccination against COVID-19 should not be mandatory.<br><br>No Confidence Intervals provided	**Score: 3**<br><br>Biased sampling frame (What was the target population? What about those without social networks?)<br><br>Measurement tool not objectively assessed (validity, reliability)<br><br>No unbiased ass6essor<br><br>Response rate not described<br><br>Refusers not described<br><br>No Confidence Intervals provided
Papini (2022) [38],<br><br>Italy	2,137	Members of the national scientific society SIMPIOS (whole population)	"e-mail invitation containing the link to the anonymous and voluntary questionnaire was sent to hospital health departments, directorates of the main health structures of the National Health Service and Local Health Units asking to forward it to the HCWs"	Online questionnaire<br><br>Measurement tool not objectively assessed (validity, reliability)	No	Response rate not described<br><br>Refusers not described	Actice HCWs<br><br>62.69% (1304/2080) of our respondents were in favor of mandatory vaccination against COVID-19<br><br>No Confidence Intervals provided	**Score: 4**<br><br>Measurement tool not objectively assessed (validity, reliability)<br><br>No unbiased ass6essor<br><br>Response rate not described<br><br>Refusers not described<br><br>No Confidence Intervals provided
Peirolo [39],<br><br>Switzerland	776	Target population: all employees of the Department of Paediatrics, Gynaecology and Obstetrics part of the University Hospitals of Geneva (whole population)	The online questionnaire was sent to all professional email addresses over a 4-week period	online questionnaire<br><br>Measurement tool not objectively assessed (validity, reliability)	No	43%<br><br>Refusers not described	Active HCWs<br><br>reasons that may change participants' mind regarding COVID-19 vaccination: Mandatory vaccination for certain situation (e.g., travel) 11/79	**Score: 4**<br><br>Measurement tool not objectively assessed (validity, reliability)<br><br>No unbiased ass6essor<br><br>Inadequate response rate

							(14%)<br><br>No Confidence Intervals provided	Refusers not described<br><br>No Confidence Intervals provided
Peruch (2022) [40],<br><br>Italy	130	Target population: HCWs of a centre for maternal and child health	Invitation was sent containing the web link to access the questionnaire<br><br>"The questionnaire was then uploaded to the intranet network of a center for maternal and child health from 4 to 31 March 2022"	online questionnaire<br><br>Measurement tool not objectively assessed (validity, reliability)	No	17.5%<br><br>Refusers not described	Active HCWs<br><br>Do you think that the mandatory vaccination of healthcare workers is right?: Yes 107 (82.3%)<br><br>No Confidence Intervals provided	**Score: 2**<br><br>Biased sampling frame (how the invitations were sent?)<br><br>Inadequate sample size (<300)<br><br>Measurement tool not objectively assessed (validity, reliability)<br><br>No unbiased ass6essor<br><br>Inadequate response rate not described<br><br>Refusers not described<br><br>No Confidence Intervals provided
Poyiadji (2022) [41],<br><br>USA	1,506	Target population: HFHS radiology department employees	-	Weekly time series vaccine compliance data	-	Response rate not described<br><br>Refusers not described	Active HCWs<br><br>A total of 14 (0.9%) radiology employees who either resigned or retired because of the vaccine mandate. Post–vaccine mandate noncompliance rates ranged from 0.5-7%.<br><br>No Confidence Intervals provided	**Score: 3**<br><br>Biased sampling frame (no description of the data collection procedure)<br><br>Unbiased ass6essor (?)<br><br>Response rate not described<br><br>Refusers not described<br><br>No Confidence Intervals provided
Qattan (2021) [42],<br><br>Saudi Arabia	736	Target population: HCWs 18 years or older and currently living in the KSA	Invitations to participate in the study were distributed to the respondents via Twitter and the WhatsApp communication platform	Online questionnaire<br><br>Measurement tool not	No	Response rate not described<br><br>Refusers not described	Active HCWs<br><br>A COVID-19 vaccine should be compulsory for all citizens and	**Score: 3**<br><br>Biased sampling frame (Those without twitter and whatsApp?)

		Simplified-snowball sampling technique		objectively assessed (validity, reliability)			residents inside Saudi Arabia: agree 248 (72.94%)<br><br>No Confidence Intervals provided	Measurement tool not objectively assessed (validity, reliability)<br><br>No unbiased ass6essor<br><br>Response rate not described<br><br>Refusers not described<br><br>No Confidence Intervals provided
Qunaibi (2021) [43],<br><br>Arab Countries	5,708	Target population: Arabic-speaking HCWs from all around the world.<br><br>Convenience sample	Social media platforms were used to conduct a digital campaign	Online questionnaire<br><br>Measurement tool not objectively assessed (validity, reliability)	No	Response rate not described<br><br>Refusers not described	Active HCWs<br><br>Only 16.2% of HCWs supported mandating the vaccine on some groups of people<br><br>No Confidence Intervals provided	**Score: 3**<br><br>Biased sampling frame (non-users of those platforms?)<br><br>Measurement tool not objectively assessed (validity, reliability)<br><br>No unbiased ass6essor<br><br>Response rate not described<br><br>Refusers not described<br><br>No Confidence Intervals provided
Regazzi (2022) [44],<br><br>Italy	2,142	Target population: Sample of Italian HCWs >18 years old	“Professional Associations and research hospitals in Northern Italy (the Maugeri Clinical Scientific Institutes), which disseminated the link to the survey through their official communication channels, such as newsletters and social media profiles. In addition, the link was disseminated through flyers during the national congress of the Italian Society of Hygiene, Preventive Medicine and Public Health.”	Online questionnaire<br><br>Measurement tool not objectively assessed (validity, reliability)	No	Response rate not described<br><br>Refusers not described	Active HCWs<br><br>The COVID-19 vaccine should be made mandatory for healthcare professionals: agree 89.5 %<br><br>No Confidence Intervals provided	**Score: 4**<br><br>Measurement tool not objectively assessed (validity, reliability)<br><br>No unbiased ass6essor<br><br>Response rate not described<br><br>Refusers not described<br><br>No Confidence Intervals provided

			The survey conducted between 22 July 2021, and 20 November 202					
Ricco (2021) [45],<br><br>Italy	166	Target population: Occupational Physicians who live and work in Italy	"OPs participating in seven different private Facebook groups and four closed forums, focusing on occupational medicine"<br><br>"To share the study invitation—the chief researcher contacted the administrators, requesting preventive authorization to post the questionnaire link, including a short description of the aims of the survey"	Online questionnaire<br><br>Measurement tool not objectively assessed (validity, reliability)	No	8.2%<br><br>Refusers not described	Active Occupational Physicians<br><br>SARS-CoV-2 vaccine should be mandatory: 60.2% agree<br><br>No Confidence Intervals provided	**Score: 2**<br><br>Inadequate sample size<br><br>Sampling (social media contact list)<br><br>Measurement tool not objectively assessed (validity, reliability)<br><br>No unbiased ass6essor<br><br>Inadequate Response rate<br><br>Refusers not described<br><br>No Confidence Intervals provided
Ruf (2022) [46],<br><br>Austria	625	Random sample for the telephone interviews, whole population for the e-mail invitation	"A list provided by the employer with all 6,033 employee work telephone numbers."<br><br>Email invitation to all employees. E-mail list not described	36 persons completed the telephone interviews<br><br>589 respondents completed the online survey<br><br>Measurement tool not objectively assessed (validity, reliability)	Interviewer<br><br>No	10% for the telephone interview<br><br>Not described for the e-mail survey invitation<br><br>Refusers not described	Nursing and social care employees<br><br>296 (64.63%) of the vaccinated and 1 (1.23%) of the unvaccinated were in favour of COVID-19 vaccine mandates.<br><br>No Confidence Intervals provided	**Score: 4**<br><br>Sampling frame (e-mail list not described)<br><br>Measurement tool not objectively assessed (validity, reliability)<br><br>No unbiased ass6essor<br><br>Inadequate response rate or not described<br><br>Refusers not described<br><br>No Confidence Intervals provided
Schmidt (2022) [47],	240	Population: 2nd–5th year dentistry	Invitation to participate in the study and a link to the online	Online questionnaire	No	66.9%	Dental students	**Score: 4**

Czech Republic		students at Charles University, Faculty of Medicine in Hradec Kralove, and Faculty of Medicine in Pilsen (the academic year 2021/2022)	questionnaire were sent to student official e-mail addresses<br><br>The online questionnaire in Google Forms could be completed from 23 May 2022 to 23 June 2022	"The test reliability was evaluated through a group of volunteer students (n = 11) who filled in the questionnaire three times in 4 weeks. For COVID-19 prevalence and COVID-19 vaccination, the Cronbach Alpha internal consistency coefficients were 1.0."		Refusers not described	75.4% of the students were in favour of vaccine mandates for the HCWs and 62.9% for the general population<br><br>No Confidence Intervals provided	Inadequate sample size<br><br>No unbiased ass6essor<br><br>Inadequate response rate<br><br>Refusers not described<br><br>No Confidence Intervals provided
Shekhar (2021) [48],<br><br>USA	3,479	A snowball sampling was utilized	"The survey tool was distributed via links posted on social media platforms in various HCW groups and distributed to administrative leaders at five major hospital systems, to disseminate among their employees"	Online questionnaire<br><br>Measurement tool not assessment not adequately (validity, reliability)	No	Response rate not described<br><br>Refusers not described	Active HCWs > 18 years old<br><br>COVID-19 Vaccine for health care workers should be mandatory: agree 35.5%<br><br>No Confidence Intervals provided	**Score: 2**<br><br>Non-random sample<br><br>Biased sampling frame (social media platforms)<br><br>Measurement tool not objectively assessed (validity, reliability)<br><br>No unbiased ass6essor<br><br>Response rate not described<br><br>Refusers not described<br><br>No Confidence Intervals provided
Singh (2021) [49],<br><br>India	254	"The survey was circulated among a closed group of HCWs chosen randomly from selected medical	Not described	Online questionnaire. For those who were not comfortable attempting	No	Response rate not described<br><br>Refusers not described	Active HCWs<br><br>Attitude towards mandatory vaccination for HCWs: 103/254 agree	**Score: 2**<br><br>Sampling frame not described<br><br>Inadequate sample size

		institutes of Uttar Pradesh "		online survey, printed copies of questionnaire were provided.<br><br>Measurement tool not objectively assessed (validity, reliability)			No Confidence Intervals provided	Measurement tool not objectively assessed (validity, reliability)<br><br>No unbiased ass6essor<br><br>Response rate not described<br><br>Refusers not described<br><br>No Confidence Intervals provided
Temsah (2021) [50],<br><br>Saudi Arabia	1,285	HCWs in Saudi Arabia<br><br>Convenience sampling technique	"Participants were invited through various healthcare providers' social media platforms, such as WhatsApp, Twitter, and emails between Dec 1 and 6, 2021"	Online questionnaire<br><br>Measurement tool assessment not adequately described	No	Response rate not described<br><br>Refusers not described	Active HCWs<br><br>The COVID-19 vaccine should be mandatory for all adult populations: agree 82.2%<br><br>No Confidence Intervals provided	**Score: 2**<br><br>Non-random sampling<br><br>Biased sampling frame (social media platforms, only 6 days available)<br><br>Measurement tool assessment not adequately described<br><br>No unbiased ass6essor<br><br>Response rate not described<br><br>Refusers not described<br><br>No Confidence Intervals provided
Tharwat (2022) [51],<br><br>Egypt	455	Target population: HCWs in Egypt<br><br>Data were gathered using convenience sampling	Early in the day, the interviewer visited the hospitals and spoke with as much HCWs as possible<br><br>From August to September 2021	"Face-to-face structured interviews were conducted by a single interviewer with each participant<br><br>five medical staff members examined the questionnaire	The interviewe r	75.8%<br><br>Refusers not described	Active HCWs<br><br>Vaccination of COVID-19 should always be compulsory once it is available: 54.3% agree<br><br>No Confidence Intervals provided	**Score: 4**<br><br>Non-random sampling<br><br>Biased sampling frame (those in absence? August to September is a day-off period for many)<br><br>Refusers not described<br><br>No Confidence Intervals provided

				design, content, wording, and simplicity of completion as part of a pilot study that validated the questionnaire"<br><br>Cronbach's alpha coefficient 0.85				
Turbat (2022) [52],<br><br>Mongolia	238	Target population: HCWs above 18 years old and currently working in public hospitals<br><br>Convenience sampling strategy	Survey invitations sent via their email and individual messaging programs (Facebook messenger) from 18 February to 23 April 2021	Online survey<br><br>Measurement tool not objectively assessed (validity, reliability)	No	15%<br><br>Refusers not described	Active HCWs<br><br>Agree that a mandatory approach to vaccination for HCWs is needed: 223/238 agree<br><br>No Confidence Intervals provided	**Score: 2**<br><br>Non-random sample<br><br>Biased sampling frame (personal or working e-mail addresses? exclusion of those without social media)<br><br>Measurement tool assessment not adequately described<br><br>No unbiased ass6essor<br><br>Response rate not described<br><br>Refusers not described<br><br>No Confidence Intervals provided
Ulbrichtova (2021) [53],<br><br>Slovakia	1,277	Target population: all HCWs and non-HCWs belonging to hospitals and general and specialised outpatient healthcare clinics in northern Slovakia	"An anonymous questionnaire was distributed to individual e-mail addresses between 30 August and 30 September 2021"	Online survey<br><br>Measurement tool not objectively assessed (validity, reliability)	No	1,277/4,268=29.9%<br><br>Refusers not described	Profession (being a physician) or/and vaccination status (being vaccinated) is an important factor towards accepting vaccine mandates<br><br>No Confidence Intervals provided	**Score: 3**<br><br>Biased sampling frame (contact list not described)<br><br>Measurement tool assessment not adequately described<br><br>No unbiased assessor<br><br>Inadequate response rate

								Refusers not described<br><br>No Confidence Intervals provided
Velikonja (2022) [54],<br><br>Slovenia	832	Target population: HCWs<br><br>Snowball sampling	"Initial group of respondents were contacted via professional (HCWs) of project members and the link to the survey was disseminated further via social network Facebook between 13 and 14 March 2020"	Online survey<br><br>Measurement tool not objectively assessed (validity, reliability)	No	7,764/18,760=41%<br><br>Refusers not described	Health sector workers<br><br>Vaccination should be mandatory for all: agree 30.6% of HCWs<br><br>No Confidence Intervals provided	**Score: 2**<br><br>Non-random sampling<br><br>Biased sampling frame (social networks and personal contacts)<br><br>Online survey<br><br>Measurement tool not objectively assessed (validity, reliability)<br><br>No unbiased assessor<br><br>Inadequate response rate<br><br>Refusers not described<br><br>No Confidence Intervals provided
Wong (2022) [55], Hong Kong, Nepal, and Vietnam	3,396	Target population:<br><br>HCWs in Hong Kong, Nepal, Vietnam	"Two rounds of email invitations were disseminated to the HCWs through the health care professional bodies and unions in Hong Kong and to major hospitals and a network of HCW voluntary consult for COVID-19 patients in Vietnam and Nepal for the recruitment. The survey was contacted in Hong Kong from May 11 to June 23 (the fourth wave); in Nepal from August 10 to November 7 (the third wave); and in Vietnam from July 12 to November 20 (the fourth wave)"	Online survey<br><br>Measurement tool not objectively assessed (validity, reliability)	No	Response rate not described<br><br>Refusers not described	Nurses and doctors aged 18 and older, working in public or private health care settings on a full-time or part-time basis Given in good health, mandatory vaccination should be applied for healthcare or residential care workers: agree 52.8%<br><br>No Confidence Intervals provided	**Score: 3**<br><br>Biased sampling frame (different sampling frames for assessing the same outcome, HCW volunteers more prone to agree with mandates)<br><br>Online survey<br><br>Measurement tool not objectively assessed (validity, reliability)<br><br>No unbiased assessor<br><br>Response rate not described<br><br>Refusers not described

								No Confidence Intervals provided
Woolf (2022) [56],<br><br>United Kingdom	3,235	Target population: HCWs participants of the UK-REACH study	"E-mail invitations were sent to 17,891 UK HCWs recruited as part of a longitudinal cohort from across the UK who had previously responded to a baseline questionnaire (primarily recruited through email) as part of the United Kingdom Research study into Ethnicity And COVID-19 outcomes in Healthcare workers (UK-REACH) nationwide prospective cohort study between 21st April and 26th June 2021"	Online survey<br><br>Questionnaire assessment described in study protocol: Woolf K, Melbourne C, Bryant L, Guyatt AL, McManus IC, Gupta A, et al. The United Kingdom Research study into Ethnicity And COVID-19 outcomes in Healthcare workers (UK-REACH): protocol for a prospective longitudinal cohort study of healthcare and ancillary workers in UK healthcare settings. BMJ Open [Internet]. 2021;11(9):e050647. Available from: https://bmjopen.bmj.com/content/bmjopen/11/9/e050647.full.pdf	No	5,633/17,891=31%<br><br>Refusers not described	Individuals aged 16 years or over, living in the UK and employed as clinical or ancillary workers in a healthcare setting and/or registered with one of seven UK professional regulatory bodies<br><br>Favours mandatory vaccination for the general population: 377 (17.9%) and for HCWs: 201 (6%)<br><br>Confidence intervals not provided (CI of OR provided but for the sum of HCWs' acceptance of COVID-19 vaccine mandates)	**Score: 6**<br><br>No unbiased assessor<br><br>Inadequate response rate<br><br>Refusers not described<br><br>No Confidence Intervals provided
Yurttas (2021) [57],<br><br>Turkey	320	Staff of university hospital (Istanbul University Cerrahpasa)	"A web-based survey link was sent out to the participants via WhatsApp link between 04 and 13 January, 2021"	Online survey<br><br>Validated questionnaire	No	22.9%	Vaccination should be mandatory: agree 35.3%<br><br>Confidence intervals not provided	**Score: 4**<br><br>Biased sampling frame (what's up contacts)<br><br>No unbiased assessor<br><br>Inadequate response rate<br><br>Refusers not described<br><br>No Confidence Intervals provided
Zheng [58],<br><br>China	618	Target population: "participants in a Jiangsu provincial symposium in obstetrics and gynaecology held in Nanjing city, the	"For the survey among the participants in the symposium, the questionnaire form was distributed to each of the participants when they entered the conference hall	Paper-based and online questionnaire<br><br>Measurement tool not objectively	No	Response rate not described<br><br>Refusers not described	HCWs at the age of 18– 60 years<br><br>2% of the vaccinated HCWs were unwilling to get vaccinated but followed the	**Score: 3**<br><br>Biased sampling frame (questionnaire available only six days for four hours. Were they informed about the

		capital of Jiangsu province and HCWs in the department of obstetrics and gynecology, Nanjing Drum Tower Hospital"<br><br>Convenience sampling	For the survey among HCWs at Nanjing Drum Tower Hospital, the questionnaire forms were distributed to each of the subjects at their convenience in the department and collected four hours later between April 5–11, 2021"	assessed (validity, reliability)			employers' mandates<br><br>Confidence intervals not provided	survey?)<br><br>Measurement tool not objectively assessed (validity, reliability)<br><br>Response rate not described<br><br>Refusers not described<br><br>Confidence intervals not provided

1. Loney PL, Chambers LW, Bennett KJ, Roberts JG, Stratford PW. Critical appraisal of the health research literature: preva- 610 lence or incidence of a health problem. Chronic Dis Can [Internet]. 1998 19(4):170–6. Available from: https://pub- 611 med.ncbi.nlm.nih.gov/10029513/
2. Aldosary AH, Alayed GH. Willingness to vaccinate against Novel COVID-19 and contributing factors for the acceptance among nurses in Qassim, Saudi Arabia. Eur Rev Med Pharmacol Sci. 2021;25(20):6386-96.
3. Aloweidi A, Bsisu I, Suleiman A, Abu-Halaweh S, Almustafa M, Aqel M, et al. Hesitancy towards covid-19 vaccines: An analytical cross–sectional study. International Journal of Environmental Research and Public Health. 2021;18(10).
4. Arif SI, Aldukhail AM, Albaqami MD, Silvano RC, Titi MA, Arif BI, et al. Predictors of healthcare workers' intention to vaccinate against COVID-19: A cross sectional study from Saudi Arabia. Saudi J Biol Sci. 2022;29(4):2314-22.
5. Badahdah AM, Al Mahyijari N, Badahdah R, Al Lawati F, Khamis F. Attitudes of Physicians and Nurses in Oman Toward Mandatory COVID-19 Vaccination. Oman Medical Journal. 2022;37(5 C7 - e429).
6. Casey SM, Burrowes SAB, Hall T, Dobbins S, Ma M, Bano R, et al. Healthcare workers' attitudes on mandates, incentives, and strategies to improve COVID-19 vaccine uptake: A mixed methods study. Hum Vaccin Immunother. 2022;18(6):2144048.
7. Ciliberti R, Lantieri F, Barranco R, Tettamanti C, Bonsignore A, Ventura F. A Survey on Undergraduate Medical Students' Perception of COVID-19 Vaccination. Vaccines (Basel). 2022;10(9).
8. Costantino C, Graziano G, Bonaccorso N, Conforto A, Cimino L, Sciortino M, et al. Knowledge, Attitudes, Perceptions and Vaccination Acceptance/Hesitancy among the Community Pharmacists of Palermo's Province, Italy: From Influenza to COVID-19. Vaccines. 2022;10(3).
9. Craxì L, Casuccio A, Amodio E, Restivo V. Who Should Get COVID-19 Vaccine First? A Survey to Evaluate Hospital Workers' Opinion. Vaccines (Basel). 2021;9(3).
10. Elbadawi MH, Altayib LS, Birier ABG, Ali LE, Hasabo EA, Esmaeel MAM, et al. Beliefs and barriers of COVID-19 vaccination hesitancy among Sudanese healthcare workers in Sudan: A cross sectional study. Hum Vaccin Immunother. 2022:2132082.
11. Fotiadis K, Dadouli K, Avakian I, Bogogiannidou Z, Mouchtouri VA, Gogosis K, et al. Factors Associated with Healthcare Workers' (HCWs) Acceptance of COVID-19 Vaccinations and Indications of a Role Model towards Population Vaccinations from a Cross-Sectional Survey in Greece, May 2021. 2021;- 18(- 19).
12. Ghirotto L, Díaz Crescitelli ME, De Panfilis L, Caselli L, Serafini A, De Fiore L, et al. Italian health professionals on the mandatory COVID-19 vaccine: An online crosssectional survey. Frontiers in Public Health. 2022;10 C7 - 1015090.
13. Giannakou K, Kyprianidou M, Christofi M, Kalatzis A, Fakonti G. Mandatory COVID19 Vaccination for Healthcare Professionals and Its Association With General Vaccination Knowledge: A Nationwide Cross-Sectional Survey in Cyprus. Frontiers in public health. 2022;10:897526.
14. Gönüllü E, Soysal A, Atıcı S, Engin M, Yeşilbaş O, Kasap T, et al. Pediatricians' COVID-19 experiences and views on the willingness to receive COVID-19 vaccines: a cross-sectional survey in Turkey. Hum Vaccin Immunother. 2021;17(8):2389-96.
15. Grabert BK, Gilkey MB, Huang Q, Yi Kong W, Thompson P, Brewer NT. Primary care professionals' support for Covid-19 vaccination mandates: Findings from a US national survey. Preventive Medicine Reports. 2022;28.
16. Jain J, Saurabh S, Kumar P, Verma MK, Goel AD, Gupta MK, et al. COVID-19 vaccine hesitancy among medical students in India. Epidemiology and Infection. 2021.
17. Jankowska-Polańska B, Sarzyńska K, Czwojdziński E, Świątoniowska-Lonc N, Dudek K, Piwowar A. Attitude of Health Care Workers and Medical Students towards Vaccination against COVID-19. Vaccines (Basel). 2022;10(4).
18. Janssen C, Maillard A, Bodelet C, Claudel AL, Gaillat J, Delory T, et al. Hesitancy towards COVID-19 Vaccination among Healthcare Workers: A Multi-Centric Survey in France. Vaccines (Basel). 2021;9(6).

19. Kashif M, Fatima L, Ahmed AM, Ali SA, Memon RS, Afzal M, et al. Perception, Willingness, Barriers, and Hesistancy Towards COVID-19 Vaccine in Pakistan: Comparison Between Healthcare Workers and General Population. 2021;-13(- 10).
20. Kaufman J, Bagot KL, Hoq M, Leask J, Seale H, Biezen R, et al. Factors Influencing Australian Healthcare Workers' COVID-19 Vaccine Intentions across Settings: A CrossSectional Survey. Vaccines (Basel). 2021;10(1).
21. Kavanagh A, Dickinson H, Dimov S, Shields M, McAllister A. The COVID-19 vaccine intentions of Australian disability support workers. Australian & New Zealand Journal of Public Health. 2022;46(3):314-21.
22. Kałucka S, Kusideł E, Głowacka A, Oczoś P, Grzegorczyk-Karolak I. Pre-Vaccination Stress, Post-Vaccination Adverse Reactions, and Attitudes towards Vaccination after Receiving the COVID-19 Vaccine among Health Care Workers. Vaccines. 2022;10(3).
23. Kelekar AK, Lucia VC, Afonso NM, Mascarenhas AK. COVID-19 vaccine acceptance and hesitancy among dental and medical students. J Am Dent Assoc. 2021;152(8):596-603.
24. Konje ET, Basinda N, Kapesa A, Mugassa S, Nyawale HA, Mirambo MM, et al. The Coverage and Acceptance Spectrum of COVID-19 Vaccines among Healthcare Professionals in Western Tanzania: What Can We Learn from This Pandemic? Vaccines. 2022;10(9 C7 - 1429).
25. Krishnamurthy K, Sobers N, Kumar A, Ojeh N, Scott A, Cave C, et al. Covid-19 vaccine intent among health care professionals of queen elizabeth hospital, barbados. Journal of Multidisciplinary Healthcare. 2021;14:3309-19.
26. Lee JT, Sean Hu S, Zhou T, Bonner KE, Kriss JL, Wilhelm E, et al. Employer requirements and COVID-19 vaccination and attitudes among healthcare personnel in the U.S.: Findings from National Immunization Survey Adult COVID Module, August - September 2021. Vaccine. 2022.
27. Lucia VC, Kelekar A, Afonso NM. COVID-19 vaccine hesitancy among medical students. Journal of Public Health. 2021;43(3):445-9.
28. Maltezou HC, Pavli A, Dedoukou X, Georgakopoulou T, Raftopoulos V, Drositis I, et al. Determinants of intention to get vaccinated against COVID-19 among healthcare personnel in hospitals in Greece. Infection, Disease and Health. 2021;26(3):189-97.
29. Maltezou HC, Rahiotis C, Tseroni M, Madianos P, Tzoutzas I. Attitudes toward Vaccinations and Vaccination Coverage Rates among Dental Students in Greece. International Journal of Environmental Research and Public Health. 2022;19(5).
30. Maltezou HC, Tseroni M, Drositis I, Gamaletsou MN, Koukou DM, Bolikas E, et al. Vaccination coverage rates and attitudes towards mandatory vaccinations among healthcare personnel in tertiary-care hospitals in Greece. Expert Review of Vaccines. 2022;21(6):853-9.
31. Mascarenhas AK, Lucia VC, Kelekar A, Afonso NM. Dental students' attitudes and hesitancy toward COVID-19 vaccine. Journal of Dental Education. 2021;85(9):1504- 10.
32. Masood FB, Nasim A, Saleem S, Jafarey AM. COVID-19 vaccine hesitancy among health service providers: A single centre experience from Karachi, Pakistan. Journal of the Pakistan Medical Association. 2022;72(6):1142-7.
33. Mayan D, Nguyen K, Keisler B. National attitudes of medical students towards mandating the COVID-19 vaccine and its association with knowledge of the vaccine. PLoS ONE. 2021;16(12 December).
34. Mustapha M, Lawal BK, Sha'aban A, Jatau AI, Wada AS, Bala AA, et al. Factors associated with acceptance of COVID-19 vaccine among University health sciences students in Northwest Nigeria. PLoS One. 2021;16(11):e0260672.
35. Navarre C, Roy P, Ledochowski S, Fabre M, Esparcieux A, Issartel B, et al. Determinants of COVID-19 vaccine hesitancy in French hospitals. Infectious Diseases Now. 2021;51(8):647-53.
36. Noushad M, Nassani MZ, Alsalhani AB, Koppolu P, Niazi FH, Samran A, et al. COVID-19 Vaccine Intention among Healthcare Workers in Saudi Arabia: A CrossSectional Survey. 2021;- 9(- 8).
37. Öncel S, Alvur M, Çakıcı Ö. Turkish Healthcare Workers' Personal and Parental Attitudes to COVID-19 Vaccination From a Role Modeling Perspective. Cureus. 2022;14(2):e22555.
38. Papini F, Mazzilli S, Paganini D, Rago L, Arzilli G, Pan A, et al. Healthcare Workers Attitudes, Practices and Sources of Information for COVID-19 Vaccination: An Italian National Survey. International Journal of Environmental Research and Public Health. 2022;19(2).
39. Peirolo A, Posfay-Barbe KM, Rohner D, Wagner N, Blanchard-Rohner G. Acceptability of COVID-19 Vaccine Among Hospital Employees in the Department of Paediatrics, Gynaecology and Obstetrics in the University Hospitals of Geneva, Switzerland. 2022;- 9.
40. Peruch M, Toscani P, Grassi N, Zamagni G, Monasta L, Radaelli D, et al. Did Italy Really Need Compulsory Vaccination against COVID-19 for Healthcare Workers? Results of a Survey in a Centre for Maternal and Child Health. 2022;- 10(- 8).
41. Poyiadji N, Tassopoulos A, Myers DT, Wolf L, Griffith B. COVID-19 Vaccine Mandates: Impact on Radiology Department Operations and Mitigation Strategies. J Am Coll Radiol. 2022;19(3):437-45.
42. Qattan AMN, Alshareef N, Alsharqi O, Al Rahahleh N, Chirwa GC, Al-Hanawi MK. Acceptability of a COVID-19 Vaccine Among Healthcare Workers in the Kingdom of Saudi Arabia. Frontiers in Medicine. 2021;8.
43. Qunaibi E, Basheti I, Soudy M, Sultan I. Hesitancy of Arab Healthcare Workers towards COVID-19 Vaccination: A Large-Scale Multinational Study. Vaccines (Basel). 2021;9(5).
44. Regazzi L, Marziali E, Lontano A, Villani L, Paladini A, Calabrò GE, et al. Knowledge, attitudes, and behaviors toward COVID-19 vaccination in a sample of Italian healthcare workers. Human Vaccines and Immunotherapeutics C7 - 2116206. 2022.

45. Riccò M, Ferraro P, Peruzzi S, Balzarini F, Ranzieri S. Mandate or Not Mandate: Knowledge, Attitudes, and Practices of Italian Occupational Physicians towards SARSCoV-2 Immunization at the Beginning of Vaccination Campaign. Vaccines (Basel). 2021;9(8).
46. Ruf AK, Völkl-Kernstock S, Eitenberger M, Gabriel M, Klager E, Kletecka-Pulker M, et al. Employer impact on COVID-19 vaccine uptake among nursing and social care employees in Austria. Front Public Health. 2022;10:1023914.
47. Schmidt J, Vavrickova L, Micopulos C, Suchanek J, Pilbauerova N, Perina V, et al. COVID-19 among Czech Dentistry Students: Higher Vaccination and Lower Prevalence Compared to General Population Counterparts. Vaccines (Basel). 2022;10(11).
48. Shekhar R, Sheikh AB, Upadhyay S, Singh M, Kottewar S, Mir H, et al. COVID-19 vaccine acceptance among health care workers in the united states. Vaccines. 2021;9(2 C7 - 119):1-18.
49. Singh AK, Kumari R, Singh S, Kandpal SD, Kaushik A. The dilemma of COVID-19 vaccination among Health Care Workers (HCWs) of Uttar Pradesh. Indian Journal of Community Health. 2021;33(2):337-42.
50. Temsah MH, Aljamaan F, Alenezi S, Alhasan K, Alrabiaah A, Assiri R, et al. SARSCoV-2 Omicron Variant: Exploring Healthcare Workers' Awareness and Perception of Vaccine Effectiveness: A National Survey During the First Week of WHO Variant Alert. Frontiers in public health. 2022;10:878159.
51. Tharwat S, Nassar DK, Nassar MK, Saad AM, Hamdy F. Attitude towards COVID-19 vaccination among healthcare workers: a cross sectional study from Egypt. BMC Health Serv Res. 2022;22(1):1357.
52. Turbat B, Sharavyn B, Tsai FJ. Attitudes towards mandatory occupational vaccination and intention to get COVID-19 vaccine during the first pandemic wave among Mongolian healthcare workers: A cross-sectional survey. International Journal of Environmental Research and Public Health. 2022;19(1).
53. Ulbrichtova R, Svihrova V, Tatarkova M, Hudeckova H, Svihra J. Acceptance of covid-19 vaccination among healthcare and non-healthcare workers of hospitals and outpatient clinics in the northern region of slovakia. International Journal of Environmental Research and Public Health. 2021;18(23).
54. Velikonja NK, Hussein M, Verdenik I, Velikonja VG, Erjavec K. COVID-19 vaccination intention at the beginning of COVID-19 pandemic in Slovenia. Zdravniski Vestnik. 2022;91(1-2):22-31.
55. Wong EL, Qiu H, Chien WT, Wong JC, Chalise HN, Hoang HT, et al. COVID-19 Vaccine Willingness and Related Factors Among Health Care Workers in 3 Southeast Asian Jurisdictions. JAMA Netw Open. 2022;5(8):e2228061.
56. Woolf K, Gogoi M, Martin CA, Papineni P, Lagrata S, Nellums LB, et al. Healthcare workers' views on mandatory SARS-CoV-2 vaccination in the UK: A cross-sectional, mixed-methods analysis from the UK-REACH study. EClinicalMedicine. 2022;46:101346.
57. Yurttas B, Poyraz BC, Sut N, Ozdede A, Oztas M, Uğurlu S, et al. Willingness to get the COVID-19 vaccine among patients with rheumatic diseases, healthcare workers and general population in Turkey: a web-based survey. Rheumatol Int. 2021;41(6):1105-14.
58. Zheng YN, Shen P, Xu BY, Chen YY, Luo YQ, Dai YM, et al. COVID-19 vaccination coverage among healthcare workers in obstetrics and gynecology during the first three months of vaccination campaign: a cross-sectional study in Jiangsu province, China.
